# Shirebi granules ameliorate acute gouty arthritis by inhibiting NETs-induced imbalance between immunity and inflammation

**DOI:** 10.1186/s13020-024-00962-6

**Published:** 2024-08-09

**Authors:** Xin Li, Xia Mao, Hong Jiang, Cong Xia, Lu Fu, Wenjing Gao, Wenjia Chen, Weijie Li, Ping Wang, Yanqiong Zhang, Haiyu Xu

**Affiliations:** State Key Laboratory for Quality Ensurance and Sustainable Use of Dao-di Herbs, Institute of Chinese Materia Medica, China Academy of Chinese Medical Sciences, Beijing, 100700 People’s Republic of China

**Keywords:** Acute gouty arthritis, Shirebi granules, Pharmacological mechanism, Neutrophil extracellular traps, Drug repurposing

## Abstract

**Background:**

Acute gouty arthritis (AGA) is classified as ‘arthritis’ in traditional Chinese medicine (TCM) theory. Shirebi granules (SGs), derived from the classic prescription SiMiaoWan, exerts satisfying therapeutic efficacy in ameliorating AGA clinically. However, the underlying mechanisms of SGs against AGA remain unclarified.

**Methods:**

AGA-related biological processes, signal pathways and biomarker genes were mined from the GEO database through bioinformatics. SGs components were systematically recognized using the UPLC-Q-TOF–MS/MS. A correlation network was established based on the biomarker genes and the chemical components, from which the signal pathway used for further study was selected. Finally, we established an AGA model using SD rats injected with monosodium urate (MSU) in the ankle joint for experimental validation. A combination of behavioral tests, H&E, safranin O- fast green, western blotting, and immunofluorescence were employed to reveal the mechanism of action of SGs on AGA.

**Results:**

The deterioration of AGA was significantly related to the imbalance between immunity and inflammation, neutrophil chemotaxis and inflammatory factor activation. HDAC5, PRKCB, NFκB1, MPO, PRKCA, PIK3CA were identified to be the candidate targets of SGs against AGA, associated with neutrophil extracellular traps (NETs) signal pathway. Animal experiments demonstrated that SGs effectively repaired cartilage damage, blocked TLR4 activation, and inhibited the expression of NETs indicators and inflammatory factors. In addition, SGs prominently alleviated joint redness and swelling, improved joint dysfunction, inhibited inflammatory infiltration of AGA rats.

**Conclusion:**

Our data reveal that SGs may effectively alleviate the disease severity of AGA by suppressing NETs-promoted imbalance between immunity and inflammation.

**Supplementary Information:**

The online version contains supplementary material available at 10.1186/s13020-024-00962-6.

## Introduction

Acute gouty arthritis (AGA) belongs to an autoimmune metabolic rheumatism with a global incidence rate of approximately 1.1% [1], which is the second most common metabolic disease in China. Epidemiological studies have shown that the prevalence of gout is increasing [2], and its prevalence in male patients is particularly high [3]. The main pathological changes associated with AGA involve congestion and edema of the joint synovium and soft tissues, accompanied by the infiltration of immune cells and exudation of proteins and cellulose. Its clinical syndromes involve the redness of joints, fever, and pain, which can impair physical mobility and decrease the quality of life of the patients if the targeted treatment is not administered on time [4]. Accumulating studies have reported that monosodium urate (MSU) in AGA can trigger the activation of macrophages, neutrophils, and monocytes, which in turn can activate innate immunity. These changes can lead to the influx of various immune cells into the joints through the secretion of immunomodulatory cytokines, which eventually result in the development of synovitis lesions in AGA patients [5, 6]. Additionally, the infiltration of neutrophils in AGA joints led to an increase in oxidative stress and the production of lipid peroxidation products through respiratory burst mechanisms. These products can directly activate pain-sensitive channels in sensory neurons that innervate the ankle joint, ultimately causing gout pain [7, 8]. Overall, the immune-inflammation imbalance and pain sensitization strongly influence the pathogenesis of AGA. Therefore, the administration of rapid anti-inflammatory and analgesic drugs is the primary strategy for treating AGA [9, 10]. However, high recurrent attacks and the risk of gastrointestinal bleeding severely hinder satisfactory therapeutic outcomes [11]. Thus, more effective drugs need to be developed to treat AGA.

In traditional Chinese medicine, AGA is classified as ‘arthritis’. Shirebi granules (SGs, National Medical License No. Z20044062), derived from the classic prescription Simiaowan, were approved by the China National Medical Products Administration in 2020 for treating arthralgia accompanied by damp-heat syndrome, and widely used in the clinical treatment of rheumatism, osteoarthritis, gout, etc. A clinical study conducted with 402 individuals showed that SGs were effective in reducing blood uric acid levels, joint pain, joint swelling, joint dysfunction and joint fever in patients with AGA [12]. Its combination with Western medicine reduced serum IFN-γ and IL-4 levels in AGA patients by 95.56% (approx.) [13]. SGs are composed of 12 Chinese herbs. Among them, *Atractylodes rhizome* and *Phellodendri chinensis cortex* are the primary drugs, *Forsythiae fructus, Dioscoreae hypoglaucae rhizome, Coicis semen* and *Stephaniae tetrandrae radix* are ministerial drugs, *Mori ramulus*, *Saposhnikoviae radix*, *Clematidis radixet rhizome, Lonice raejaponicae caulis, pheretima and Cyathulae radix* are used as adjuvant drugs. The combination of these drugs helps eliminate wind and dampness, clear heat and reduce swelling, clear collaterals and relieve pain. Although the clinical efficacy of SGs in treating AGA is high, research on its mechanism of action is limited. This lack of information prevents the widespread use of this treatment agent.

In this study, we comprehensively mined GEO microarrays by performing gene set enrichment analysis (GSEA), and weighted gene co-expression network analysis (WGCNA) to investigate AGA-related signaling and genes. Then, the components of SGs were identified using the LC–MS/MS technique, and the protein–protein interaction (PPI) network was established using the targets of SGs and the gene sets of AGA-related pathological modules. Next, potential biomarkers and the molecular regulation of SGs associated with the treatment of AGA were further assessed, and an immune infiltration analysis was performed. The AGA rat model was used to experimentally verify the pharmacological efficacy and to elucidate the mechanisms of SGs against AGA.

## Materials and methods

### Bioinformatics analysis of AGA

#### Microarray dataset acquisition

The gout gene microarray datasets of AGA were retrieved from the GEO database (https://www.ncbi.nlm.nih.gov/geo) using the keywords "Gouty arthritis" or "Gout", Selecting the species as "Homo sapiens" or "Mus musculus", three datasets, including GSE160170, GSE178825, and GSE190138, were obtained. Both GSE178825 and GSE160170 utilized human peripheral blood mononuclear cells (PBMC) as the research tissue, and GSE190138 used C57 mouse ankle joint, dorsal root ganglion (DRG), and spinal cord as research tissues.

#### Data processing and identification of differentially expressed genes

Network Analyst3.0 (https://www.networkanalyst.ca/) [14], an online data analysis website based on R language, was used to screen differential expressed genes (DEGs) in the AGA and control groups. The DEGs were screened based on the criteria *P* < 0.05 and |log2FC|> 1.5.

#### Pathway enrichment analysis

Signal pathway enrichment of DEGs was performed based on Gene Ontology (GO, http://www.geneontology.org) [15] and the Kyoto Encyclopedia of Genes and Genomes (KEGG, http://www.genome.jp/kegg/) database [16]. The DEGs were imported into the DAVID database (DAVID 6.8, http://david.ncifcrf.gov) [17] and *P* < 0.05 was considered to be statistically significant. The GSEA included all genes in the dataset and helped to select significantly enriched sets of genes, using the R package "clusterProfiler". The top 10 enrichment results were visualized using the "ggplot2" package in R, with *P* < 0.05 as the screening criterion.

#### WGCNA

Weighted gene co-expression network analysis of co-expressed genes was performed using the WGCNA package in the R software [18]. The pickSoft threshold function was used to estimate a suitable soft threshold β, which was used to construct a network that fitted the characteristics of a scale-free network. Hierarchical clustering was performed using the ‘hclust’ function, and the dynamic tree cut algorithm was used to identify gene modules. Next, gene significance (GS) and module membership (MM) were calculated and the modules were associated with clinical traits by setting the parameters |MM|> 0.8 and |GS|> 0.1 to screen for genes.

### Identification of the composition SGs

#### Materials and reagents

Shirebi granules (SGs; Lot. 210 602) was obtained from Liaoning Good Nurse Pharmaceutical (Group) Co., Ltd. The composition ratio of SGs included 108 g of *Atractylodes rhizome* [from *Atractylodes lancea* (Thunb.) DC.], 108 g of *Phellodendri chinensis cortex* [from *Phellodendron chinense* C.K.Schneid.], 162 g of *Forsythiae fructus* [from *Forsythia suspensa* (Thunb.) Vahl]*,* 162 g of Dioscoreae *hypoglaucae rhizome* [from *Dioscorea hypoglauca* Palibin]*,* 216 g of *Coicis semen* [from *Coix lacryma-jobi* L.var.*ma-yuen* (Roman.) Stapf], 162 g of *Stephaniae tetrandrae radix* [from *Stephania tetrandra* S.Moore], 216 g of *Mori ramulus* [from *Morus alba* L.], 108 g of *Saposhnikoviae radix* [from *Saposhnikovia divaricata* (Turcz.) Schischk.], 129 g of *Clematidis radixet rhizome* [from *Clematis chinensis* Osbeck]*,* 216 g of *Lonice raejaponicae caulis* [from *Lonicera japonica* Thunb.]*,* 108 g of *Pheretima* [from *Pheretima aspergillum* (E.Perrier)] and 162 g of *Cyathulae radix* [from *Cyathula officinalis* K.C.Kuan]. The names of the medicinal materials were checked using http://www.worldfloraonline.org on April 20, 2024. The SGs were prepared as follows: first, volatile oils were extracted from *Atractylodes rhizome*, *Forsythiae fructus* and *Saposhnikoviae radix*. The remaining nine herbs were decocted with water to obtain an extract, which was filtered and concentrated to form a fluidized ointment. After adding ethanol thrice, the ointment was left undisturbed for 12 h. The resulting supernatant was concentrated to form a clear paste with a relative density of 1.33–1.35, measured at 50 °C. After drying, SGs were prepared by spraying the volatile oils extracted above. Methanol (SN. 1.06 007.4 008, Lot. I1228 607 234) and acetonitrile (SN. 1.00 030.4 008, Lot. JB123 830) were purchased from Merck.

#### Sample preparations and solutions

0.5 g of SGs powder was accurately weighed and placed in a stoppered Erlenmeyer flask. Next, 50 mL of 70% methanol was accurately added to the flask and covered with the stopper. The total mass of the powder and stoppered flask was then weighed. The SGs powder was then ultrasonically extracted for 30 min, followed by cooling of the extract. After centrifuging the extract at 12,000*g* for 10 min, the supernatant of the extract was collected, filtered through a 0.22 µm PTFE filter membrane (Agilent Technologies, USA), and stored at 4 °C.

#### UPLC-Q-TOF–MS/MS analysis

The compositional analysis of SGs was based on the pre-established method [19]. UPLC-Q-TOF–MS/MS analysis was performed using an ACQUITY UPLC-Q-TOF System (Waters USA). Samples were analyzed using Waters Acquity UPLC HSS T_3_ column (2.1 × 100 mm 1.8 μm; Waters, USA) at 40 °C. The mobile phase composed of 0.1% (*v/v*) formic acid in water (A) and acetonitrile (B), was analyzed at a flow rate of 0.5 mL/min. A gradient elution program was used as follows: 0–8 min, 2–10% B; 8–14 min, 10–15% B; 14–20 min, 15–20% B; 20–25 min, 20–40% B; 25–28 min, 40 – 98% B; 28–31 min, 98% B. The injection volume was 2 μL.

Mass spectrometry was performed using a Waters SYNAPT G2HDMS System (Waters Corp.) equipped with an electrospray ionization (ESI) source in both positive and negative ion modes. The capillary voltage was 2.6 kV, cone voltage was 40 V, desolvation AGAs (N_2_) flow rate was 800 L/h, and source temperature and desolvation temperature were set at 120 °C and 400 °C, respectively. The trap collision energy was increased from 10 to 30 eV. All data were acquired and processed using the Masslynx 4.1 software (Waters).

### Protein–protein interaction network analysis and construction of a compound-target-pathway-disease network

ETCM v2.0 (http://www.tcmip.cn/ETCM2/front/#/browse/herb) [20] and TCMIP v2.0 (http://www.tcmip.cn/TCMIP/index.php/Home) [21] platforms were used to predict the targets for the components present in SGs. A protein–protein interaction network (PPI network) was constructed based on the String database (http://cn.string-db.org/) [22] for the targets of SGs and gene sets of AGA, which were obtained from WGCNA. The top 20 key targets were identified by the MCC algorithm of CytoHubba in the Cytoscape3.7.1 software. To further elucidate the mechanisms of SGs against AGA, the ‘component-target-pathway-disease’ interaction network was constructed using the links of potentially active ingredients, key targets, and key pathways related to the treatment of AGA with SGs.

### Immune-infiltration analysis

The CIBERSORT algorithm (http://cibersort.Stanford.edu/) in the R software was used to calculate the relative proportions of 22 immune infiltrating cells in both the peripheral blood and ankle joints. Next, the Wilcoxon rank sum test was performed to compare the differences in the content of immune cells between the AGA group and the control group. The screening criterion was *P* < 0.05. To assess the relationship between the key genes involved in the treatment of AGA with SGs and immune cells, Spearman’s correlation analysis was performed.

### Animal care and experiments

#### Animals

A total of 60 male SD rats of specific-pathogen-free grade, 6–8 weeks, weighing between 200 and 220 g were purchased from Beijing Vital River Laboratory Animal Technology Co., Ltd. (animal license number: SCXK (Jing) 2019-0008). During the experimental period, all rats were provided free access to water and solid chow and were maintained at constant temperature and humidity in the animal facility of the Institute of Traditional Chinese Medicine, China Academy of Traditional Chinese Medicine. All experimental protocols were approved by the Animal Experiment Ethics Committee of the Institute of Chinese Materia Medica, China Academy of Chinese Medical Sciences. The experiments were performed strictly following the Guidelines for the Care and Use of Laboratory Animals of the National Institutes of Health (Ethics number: 2022B067).

#### Treatment groups

SD rats were randomly divided into six groups (n = 10 rats per group) after adaptive feeding for one week. These groups included control (Con), model (AGA), low-dose treatment (SGs, 2.5 g/kg), medium-dose treatment (SGs, 5 g/kg), high-dose treatment (SGs, 10 g/kg) and positive drug treatment (Colchicine, 0.3 mg/kg). The low, medium and high-dose of SGs were equivalent to one, two and four times the clinical equivalent dose, respectively. After 5 d of gavage administration, a 100 μL solution of 5 μg MSU was injected into the ankle joint cavity of the rats in all groups except for the control group. Then, the treatment was administered for two more days.

#### Sample collection

On 7 d, all rats were anesthetized with 2% isoflurane 1 h after administration and blood was collected from the abdominal artery. The collected blood samples were left in a vacuum blood collection tube for 30 min, followed by centrifugation at 3000×*g* for 15 min, serum was collected and stored at − 80 °C. After blood samples were collected, the rats were euthanized, and the ankle joint area was examined from 0.5 cm above to 0.5 cm below. The skin and surrounding muscle tissue were peeled off within 1 min. The ankle joints of the rats from each group were fixed with 4% paraformaldehyde to prepare histopathological synovial sections and perform immunofluorescence detection. The remaining ankle tissue was stored at − 80 °C for further use.

### Assessment of arthritis

To assess the effects of SGs on the ankle joint dysfunction of rats, an ankle disability index scale was used 24 h after modeling. The scoring criteria for were as follows [23]: 0 points indicated normal walking, 1 point indicated slightly bent lower limbs and lameness, 2 points indicated moderate lameness with the lower limbs only touching the ground, and 3 points indicated trigeminal walking with severe lameness. Additionally, an ankle joint inflammatory index was established and interpreted based on the following criteria [24]: 0 points indicated normal joints, 1 point indicated visible bone markings and mild joint redness and swelling, 2 points indicated non-visible bone markings and visible joint redness and swelling, and 3 points indicated redness and swelling in extra-articular limbs, such as the thigh. The circumference of the right ankle joint [25] was also measured before and 24 h after modeling using digital calipers with a minimum accuracy of 0.01 mm. The swelling index was calculated using the formula Swelling index = (ankle circumference before modeling - ankle circumference after modeling)/ankle circumference before modeling. The measurement was repeated three times for each group and the average value was recorded.

### Paw withdrawal threshold and thermal pain threshold

AGA is often accompanied by intense pain, and evaluating the pain threshold can provide insights into the onset and medication effects of the disease [26]. To investigate mechanical hyperalgesia, von Frey hair tests were performed [27]. The measurements were performed on d 4 and d 6, and each measurement was maintained for three cycles with an interval of 5 min, and the average value was calculated.

To investigate thermal hyperalgesia in rats, the YLS-6B intelligent hot plate instrument was used to measure the thermal pain threshold [28] on 4 d and 6 d. The rats were placed in a hot plate apparatus at (55 ± 0.5) °C, and the time (s) from the moment the rats touched the hot plate to the reaction of licking the hind paw was considered to be the pain threshold of the rat. Each measurement was maintained for 3 cycles with an interval of 30 min, and the average value was calculated.

### Enzyme-linked immunosorbent assay

After thawing the rat serum samples at room temperature, the levels of interleukin-1beta (IL-1β, Cat# MM-0 047R1), interleukin-6 (IL-6, Cat# MM-0 190R1), tumor necrosis factor alpha (TNF- α, Cat# MM-0 180R1), citrullinated histone H3 (CitH3, Cat# MM-71 581R2), neutrophil elastase (NE, Cat# MM-0 306R1) and myeloperoxidase (MPO, Cat# MM-50 254R2) were detected using the ELISA kit (MEIMIAN, Jiangsu, China) following the manufacturer’s instructions.

### H&E and Safranin O/Fast Green staining

The ankle joint tissues of the rats were fixed in 4% paraformaldehyde, decalcified using ethylene diamine tetraacetic acid (EDTA), dehydrated stepwise with ethanol, made transparent with xylene, embedded in paraffin, and sliced into thin sections (2–4 μm thick). The ankle tissue sections were stained with hematoxylin for 10 min, re-stained with eosin (Cat#G1120, Solarbio, Beijing, China), and sealed. Then, the morphological and pathological changes in the synovial tissue of the ankle were observed under a microscope at 5× and 20× magnification (scale bar: 200 μm and 50 μm).

The ankle joint tissue sections were dipped in Fast Green staining solution for 5 min, washed with a weak acid solution, and dipped in Safranin O stain (Cat#G1371, Solarbio, Beijing, China) for 5 min. The sections were examined under a microscope at 5× and 20× magnification, and images were acquired and analyzed (scale bar: 200 μm and 50 μm). A modified Mankin's method [29] was used to evaluate the degree of cartilage degeneration in rats; a higher score indicated, more serious degeneration.

### Western blotting

Ankle tissues were minced and incubated with radioimmunoprecipitation assay (RIPA) lysate for 30 min. After incubation, the contents were centrifuged, and the resulting protein supernatant was collected and quantified using the BCA method. Next, 30 μg of protein underwent SDS-PAGE, and then, it was transferred to a PVDF membrane, the membrane was blocked with skim milk for 1.5 h at room temperature. Primary antibodies against TLR4 (1:1 000, Cat# sc-293 072, Santa Cruz Biotechnology, USA), PKC (1:1 000, Cat# 9 616, Cell Signaling Technology, Beverly, MA), CitH3 (1:1 000, Cat# ab5 103, Abcam, Cambridge, United Kingdom), and MPO (1:4 000, Cat# 66 177–1-Ig, Proteintech, Wuhan, China) were incubated with the membranes overnight at 4 °C. The following day, secondary antibodies were added and incubated at room temperature for 1 h. The relative protein expression was quantified using the ImageJ software after chemiluminescence development.

### Immunofluorescence

The paraffin sections of the ankle joints of rats were dewaxed and then antigen retrieval was performed in a microwave oven with sodium citrate buffer (pH 8.0). After the sections were blocked with BSA at room temperature for 30 min, the primary antibodies MPO (1:500) and CitH3 (1:1 000) were added and incubated overnight at 4 °C in a humid box. The following day, secondary antibodies were added and incubated at room temperature for 30 min. The nuclei were counterstained with DAPI and mounted with an anti-fluorescence quencher. The samples were observed, and images were captured using a confocal microscope.

### Statistical analysis

All data were analyzed using the SPSS 26.0 software, and graphs were plotted using the GraphPrism 9 software. To ensure that the data were accurate and reliable, biological replicates were performed with six rats per group and all experiments were conducted thrice, the results were reported as the mean ± SEM. The data were assessed for normality using the Shapiro–Wilk test and for variance using the Chi-square test. Afte the requirements were met, the differences in parameters among groups were analyzed by performing one-way ANOVA. When the data did not follow a normal distribution or had unequal variances, the differences in parameters among groups were compared by the Kolmogorov–Smirnov nonparametric test. All differences among and between groups were considered to be statistically significant at* P* < 0.05.

## Results

### Main pathological changes associated with AGA progression

#### DEGs were screened from GEO datasets

**Fig. 1:**
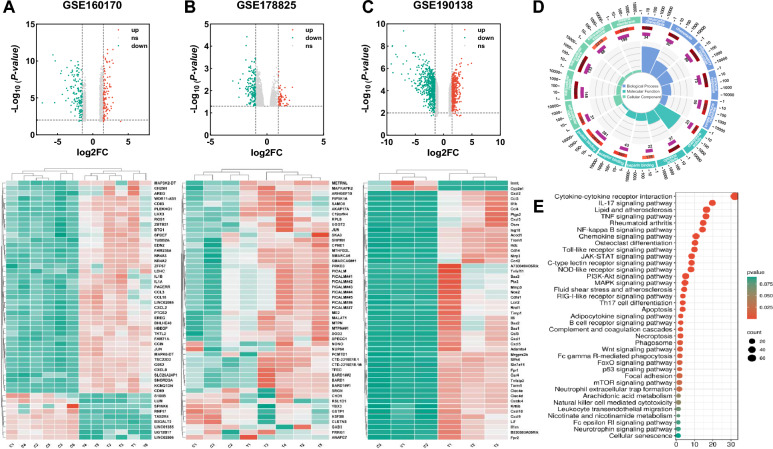
Identification of differentially expressed genes (DEGs) and functional enrichment analysis of DEGs. **A** Volcano plots and heatmap of DEGs in GSE160 170; **B** Volcano plots and heatmap of DEGs in GSE178 825; **C** Volcano plots and heatmap of DEGs in GSE190 138. Volcano plots showed the DEGs by the criteria of |log2FC|> 1.5 and *P* < 0.05. The up-regulated genes were marked in red, while the down-regulated genes were marked in green. The top 50 DEGs with the largest |log2FC| are shown in the heatmap. **D** The results of GO enrichment categories included biological process (BP), cellular component (CC), and molecular function (MF). **E** The results of KEGG pathway enrichment analyses of the DEGs

DEGs were identified from GSE160 170, GSE178 825, and GSE190 138 datasets. The GSE160 170 chip showed 246 DEGs, including 139 downregulated genes and 107 upregulated genes (Fig. 1A). The GSE178 825 chip contained 176 DEGs, including 118 downregulated genes and 58 upregulated genes (Fig. 1B). The GSE190 138 chip contained 1 045 DEGs, including 611 downregulated genes and 434 upregulated genes (Fig. 1C). Three heatmaps were plotted using the top 50 DEGs with the highest |log2FC| values in each chip.

GO and KEGG enrichment analysis of DEGs showed that they were mainly involved in the inflammatory immune response, such as innate immune response, neutrophil chemotaxis and inflammatory factor activation (Fig. 1D). The deterioration of AGA was related to abnormalities in TNF signaling, NF-kappa B signaling, chemokine signaling, neutrophil extracellular traps etc*.* (Fig. 1E).

#### *Inflammatory-immune imbalance and abnormal energy metabolism were the key pathological links involved in AGA as annotated *via* GSEA*

**Fig. 2:**
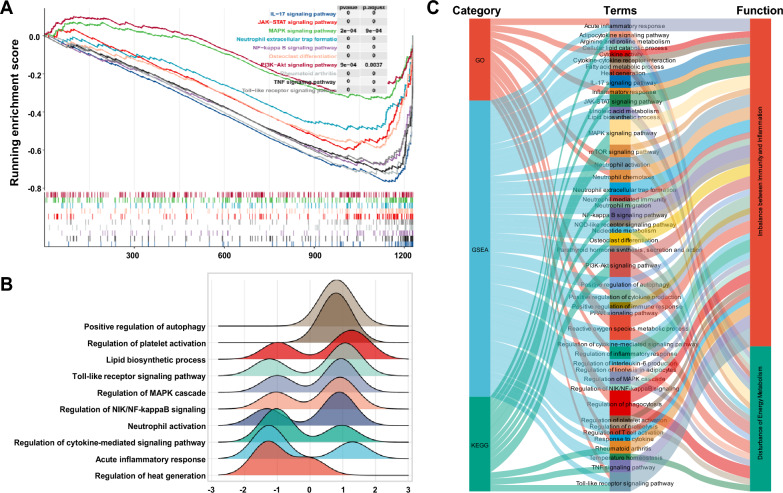
GSEA results based on GO and KEGG databases. **A** 10 representative enriched GSEA-KEGG pathways. **B** 10 representative enriched GESA-GO gene sets. **C** Sankey diagram for GO, KEGG and GSEA

We performed GSEA analysis on the GEO dataset genes to identify unique pathological links of AGA. The GSEA-KEGG results showed that the AGA genes were mainly enriched in IL-17, Toll-like receptor, NF-kappa B, neutrophil extracellular traps, TNF, PI3K-Akt and MAPK signal pathway. (Fig. 2A). The results of the GSEA-GO analysis showed that the AGA genes were significantly enriched in Toll-like receptor, neutrophil activation, acute inflammatory response, positive regulation of autophagy, lipid biosynthetic process, etc. (Fig. 2B). Compared to GSEA, the GO and KEGG enrichment analysis conducted using DAVID focused on identifying inflammation-immune system imbalances. The GSEA included additional pathways such as linoleic acid metabolism, regulation of lipolysis in adipocytes, and PPAR signaling, along with inflammation-immune system imbalance pathways. These pathways were mostly related to disturbances in material and energy metabolism, which was consistent with the characteristics of AGA as a metabolic disease (Fig. 2C).

#### Identification of AGA-related key genes by WGCNA

**Fig. 3:**
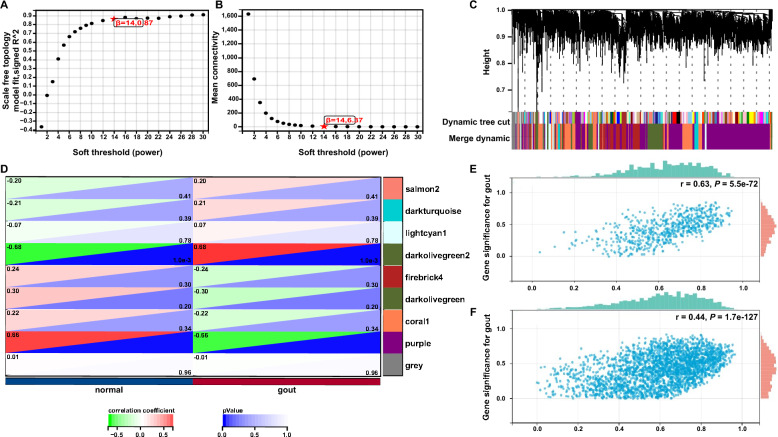
Construction and analysis of gene co-expression network. **A** Analysis of the scale-free index for various soft-threshold powers (β). **B** Analysis of the mean connectivity for various soft-threshold powers. **C** Shows the original and combined modules under the clustering tree. **D** Heatmap of the correlation between the module eigengenes and clinical traits of AGA. **E** Scatter plot of gene significance in darkolivegreen2 module. **F** Scatter plot of gene significance in purple module

In WGCNA, a soft threshold of 14 and a minimum module value of 30 were used to define the adjacency matrix (Fig. 3A and B). The gene tree constructed by the hierarchical clustering of gene neighborhood coefficients is shown in Fig. 3C. Two key gene modules (ME darkolivegreen2, ME purple) with 501 genes were found to be closely related to the development of AGA through the association between modular genes and disease (Fig. 3D). The correlation analysis between GS and MM of the key modules showed that the strongest correlation was between darkolivegreen2 module and with AGA (r = 0.63, *P* = 5.5e−72, Fig. 3E), followed by the correlation between the purple module and AGA (r = 0.44, *P* = 1.7e−127, Fig. 3F). Therefore, the genes in the darkolivegreen2 and purple modules were integrated as key gene sets for AGA in the subsequent analysis.

### Determining the potential mechanisms of SGs in the treatment of AGA

#### Chemical profiling of SGs identified by UPLC-Q-TOF/MS

**Fig. 4:**
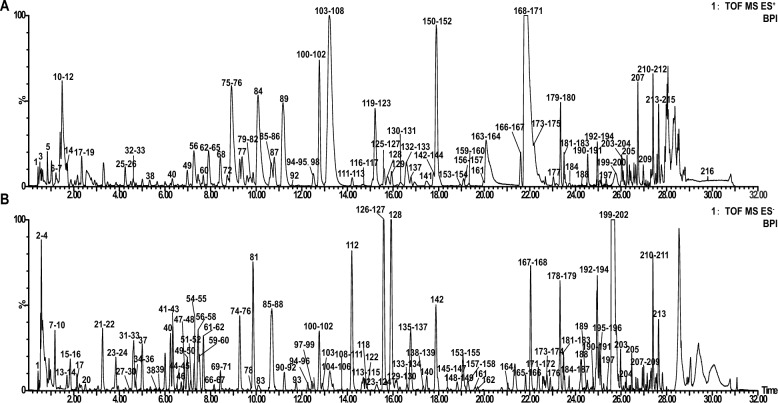
Base peak ion (BPI) chromatograms of SGs in UPLC-Q-TOF–MS. **A** BPI diagram in positive ion mode. **B** BPI diagram in negative ion mode

BPI diagrams were obtained in positive and negative ion modes (Fig. 4). The results showed that 216 compounds were tentatively identified in SGs, including 128 compounds in the positive ion mode and 169 compounds in the negative ion mode. Information regarding the 216 compounds is outlined in Additional file 1: Table S1 and Table S2.

#### SGs may ameliorate AGA by suppressing NETs-promoted imbalance between immunity and inflammation

**Fig. 5:**
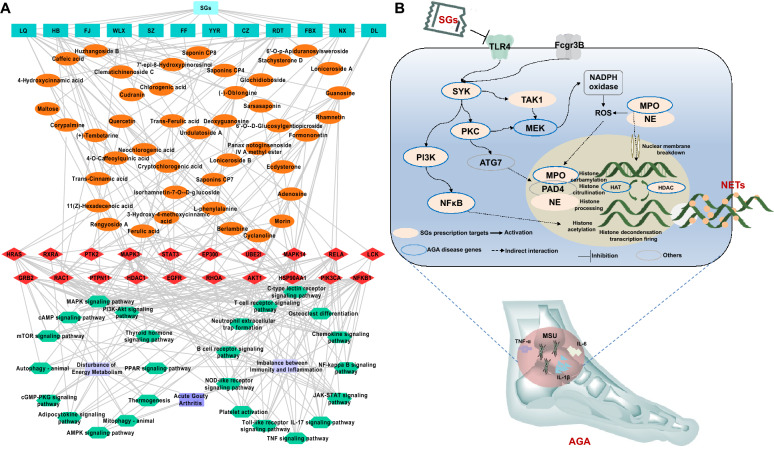
Network analysis indicated that SGs may ameliorate AGA by suppressing NETs-promoted immunity-inflammation imbalance. **A** The component-target-pathway network associated with the pharmacological mechanisms of SGs against AGA. LQ: *Forsythiae fructus*; HB: *Phellodendri chinensis cortex*; FJ: *Stephaniae tetrandrae radix*; WLX: *Clematidis radixet rhizome*; SZ: *Mori ramulus*; FF: *Saposhnikoviae radix*; YYR: *Coicis semen*; CZ: *Atractylodes rhizome*; RDT: *Lonice raejaponicae caulis*; FBX: *Dioscoreae hypoglaucae rhizome*; NX: *Cyathulae radix*; DL: *Pheretima*. **B** Illustration of SGs putative targets involved into NETs-promoted imbalance between immunity and inflammation

To investigate the potential mechanisms of SGs against AGA, 1 088 genes were predicted as the SGs putative targets of 216 chemical components identified in “UPLC-Q-TOF-MS/MS analysis” section Then, the PPI network was established between the key gene sets related to AGA obtained from WGCNA and the SGs putative targets. After identifying the direct links between AGA-related key genes and SGs putative targets, the functional enrichment analysis was performed, and the results indicated that the therapeutic targets of SGs against AGA were significantly associated with NETs, PI3K/AKT, NF-κB, AMPK, TNF, and PPAR signal pathways*.* (Fig. 5A). Among them, NETs signaling is associated with immune response and plays a crucial role in the pathogenesis of AGA [30]. Notably, six putative targets of SGs, including TLR4, SYK, PI3K, PKC, NF-κB, MPO, and NE, were found to participate in NETs signaling (Fig. 5B). It has been reported that TLR4 may be recognized by MSU, which is the pathological product of AGA, leading to the activation of SYK and triggering of the innate immune response [31], these changes can facilitate the recruitment of neutrophils to the site of the lesion [32]. TLR4/SYK activation may trigger the activation of PI3K/NF-κB, resulting in the production of inflammatory factors such as IL-1β [33]. After the accumulation of these inflammatory factors in neutrophils, the massive transcription and translation of NE, MPO and PAD4 may enter the nucleus, within which, PAD4 citrullinated nuclear histones, causing chromatin depolymerization and dissolution of the nuclear and granule membranes. Subsequently, a large number of antibacterial proteins in the cytoplasm bound to the depolymerized chromatin, forming a network structure that was eventually released extracellularly as NETs [34], which may induce a continuous production of inflammatory factors in tissues, thus exacerbating the production of NETs. Moreover, MSU has been indicated to activate autophagy through SYK/PKC in vivo [35], and autophagy, in turn, can enhance the formation of NETs [36]. Interestingly, SYK and PKC influence NADPH oxidase to generate a substantial amount of ROS, promoting neutrophil lysis and the subsequent formation of NETs [37, 38]. On the basis of these findings, we hypothesized that SGs might suppress the formation of NETs, subsequently modulating the inflammatory response during the progression of AGA.

#### SGs putative targets involved into NETs signaling may be associated with neutrophil infiltration into the ankle site of AGA

**Fig. 6:**
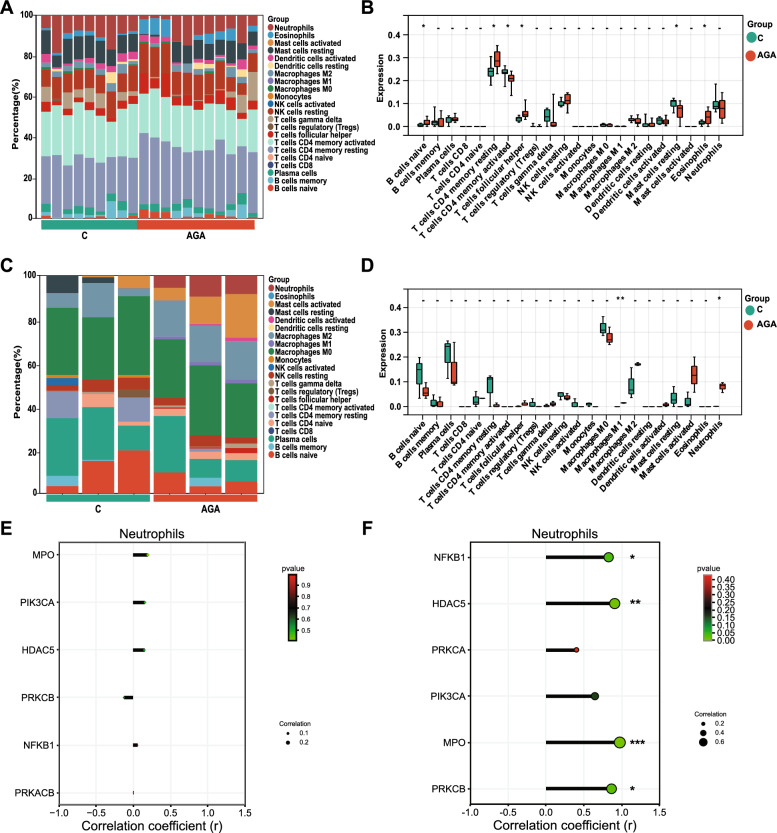
Immune-infiltration analysis. **A** The relative proportion of 22 types of immune infiltrating cells in peripheral blood samples of C and AGA patients is shown as a barplot. **B** Box plot of the abundance of each type of immune cell infiltration in peripheral blood samples of C and AGA groups. **C** The relative proportion of 22 types of immune infiltrating cells in the ankle joint samples of C and AGA patients is shown as a barplot. **D** Box plot of the abundance of each type of immune cell infiltration in the ankle joint samples of C and AGA groups. **E** The correlation between 6 key genes and neutrophils in peripheral blood samples. **F** The correlation between 6 key genes and neutrophils in ankle joint samples

An immune infiltration analysis was performed to determine the immune response during the development of AGA. The CIBERSORT analysis tool was used to calculate the distribution ratios of 22 kinds of immune cells in the blood and the inflamed ankle joints in AGA and normal control groups. The difference in the distribution ratio of immune cells between the AGA group and the normal group was significant (*P* < 0.05, Fig. 6A and C). Notably, the number of neutrophils was significantly higher in the ankle joints of patients with AGA (*P* < 0.05, Fig. 6B and D), which was different from the distribution of neutrophils in the peripheral blood. Moreover, all six SGs putative target genes (HDAC5, PRKCB, NFκB1, MPO, PRKCA, and PIK3CA) were involved into the formation of NETs in the AGA group (Fig. 6E and F).

### Experimental Validation

#### SGs administration alleviates the severity of AGA

**Fig. 7:**
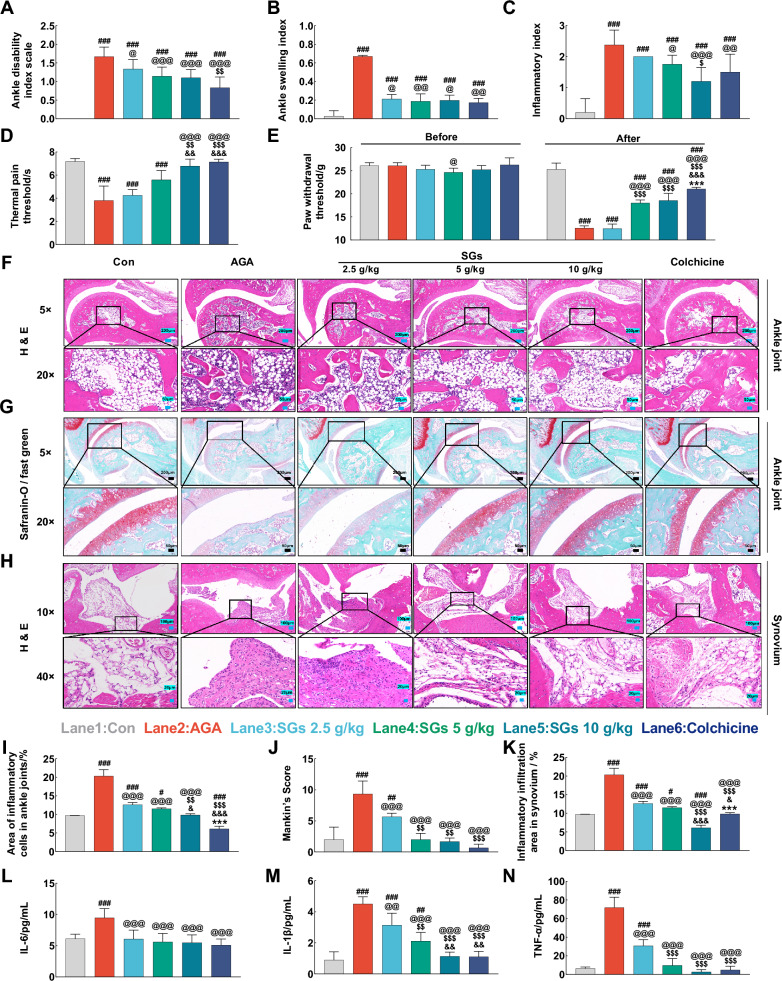
Improvement effects of SGs on the severity of arthritis in AGA rats. **A** Ankle disability index scale. **B** Ankle swelling index. **C** Inflammatory index. **D** Thermal pain threshold. **E** Paw withdrawal threshold. **F** Hematoxylin and eosin (H&E) staining of ankle joints. Scale bar, 200 μm and 50 μm. **G** Safranin-O/fast green staining of ankle joints. Scale bar, 200 μm and 50 μm. **H** H&E staining of synovium. Scale bar, 100 μm and 20 μm. **I** Area of inflammatory cells in ankle joints. **J** Mankin’s score. **K** Inflammatory infiltration area in synovium. **L** IL-6. **M** IL-1β. **N** TNF-α. n = 6, $$\overline{x}$$  ± *s*, ^#^*P* < 0.05, ^##^*P* < 0.01, and ^###^*P* < 0.001 vs. Con; ^@^*P* < 0.05, ^@@^*P* < 0.01, and ^@@@^*P* < 0.001 vs. AGA; ^$^*P* < 0.05, ^$$^*P* < 0.01, and ^$$$^*P* < 0.001 vs. 2.5 g/kg SGs; ^&^*P* < 0.05, ^&&^*P* < 0.01, and ^&&&^*P* < 0.001 vs. 5 g/kg SGs; ^*^P < 0.05, ^**^P < 0.01, and ^***^P < 0.001 vs. 10 g/kg SGs

The ankle disability index, ankle swelling index and inflammatory index were significantly higher (*P* < 0.001), whereas the thermal pain threshold and paw withdrawal threshold were significantly lower (*P* < 0.001) in the AGA model group compared to that in the control group. Administering low, medium, and high doses of SGs significantly reduced the ankle disability index (Fig. 7A), ankle swelling index (Fig. 7B), and inflammatory index (Fig. 7C). SGs also considerably increased the thermal pain threshold (Fig. 7D) and paw withdrawal threshold (Fig. 7E). Treatment with medium and high doses of SGs caused no significant difference in the ankle disability index, ankle swelling index, or inflammatory index compared to their respective values in the colchicine group. The improvement effect recorded in the high-dose of SGs group on thermal pain threshold was similar to that recorded in the colchicine group.

The H&E-stained sections showed that the ankle joint structure of the AGA rats was severely disrupted, the infiltration of inflammatory cells increased significantly (*P* < 0.001) synovial cells were disorganized, and cell proliferation was altered, compared to the ankle joint structure of the rats in the control group. In addition, the area of inflammatory cell infiltration in the synovium of the rats in the AGA group was significantly greater than that in the rats of the Con group (*P* < 0.001). The treatment with three doses of SGs repaired the damaged structure of the ankles (Fig. 7F), accompanied by a reduction in the number of inflammatory cells that infiltrated the site affected by inflammation in the ankle joints and the synovium (Fig. 7H, I, K).

The rats in the AGA model group exhibited a disorganized hierarchy of cartilage structure, lighter staining of the cartilage matrix, and a smaller extent of red staining compared to that recorded in the rats of the control group. These results suggested a reduction in the articular cartilage matrix, disruption of cartilage, poorly defined intact joint gaps, and formation of osteoclasts, which were consistent with Mankin’s score (Fig. 7J). SGs treatment effectively relieved cartilage damage in ankle joints (Fig. 7G), inhibited osteoclast formation, and restored tissue structure in the articular cartilage and subchondral bone.

The levels of the inflammatory factors IL-6, IL-1β, and TNF-α in serum were significantly higher (*P* < 0.01) in the AGA rats. Treatment with three doses of SGs restored the level of expression of serum IL-6 (*P* < 0.001, Fig. 7L), IL-1β (*P* < 0.05, Fig. 7M), and TNF-α (*P* < 0.001, Fig. 7N). The difference in efficacy between the high dose of SGs group and the colchicine group was not significant.

#### *SGs inhibits the NETs formation in AGA *via* TLR4/PKC signaling*

**Fig. 8:**
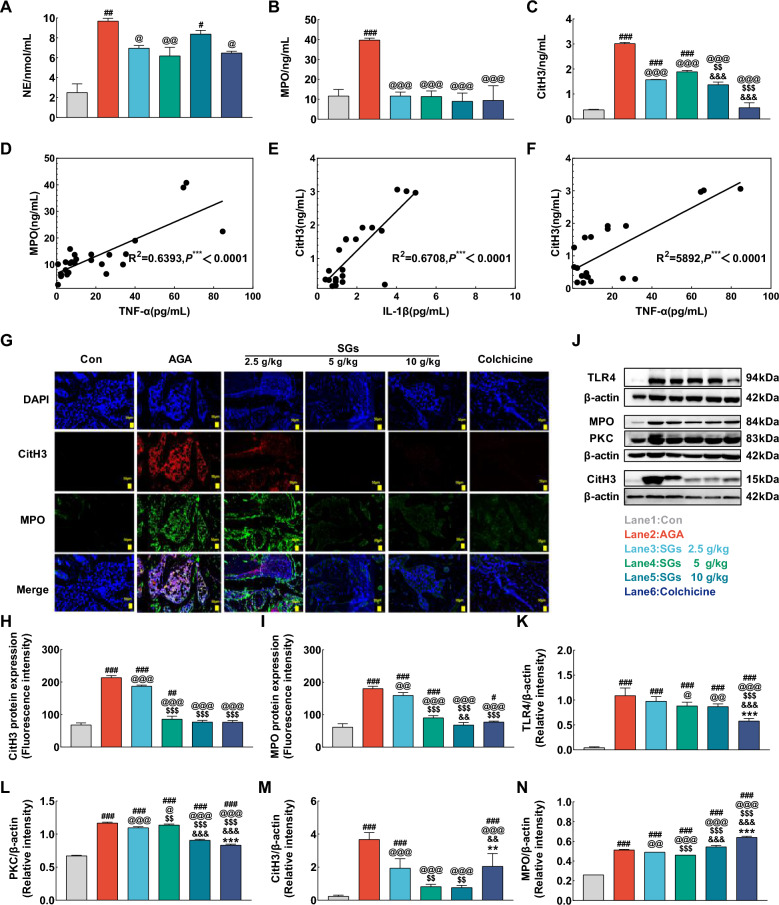
The regulatory effects of SGs on the expression of proteins involved into TLR4/PKC/NETs signaling of AGA rats. **A** Serum content of NE. **B** Serum content of MPO. **C** Serum content of CitH3. **D** Correlation between serum contents of MPO and TNF-α. **E** Correlation between serum contents of CitH3 and IL-1β. **F** Correlation between serum contents of CitH3 and TNF-α. **G** Immunofluorescence staining of NETs formation in the ankle joint of AGA rats stained with DAPI (blue), anti-CitH3 (red), and anti-MPO (green) antibodies. Scale bar, 50 μm. **H** The relative fluorescence intensities of CitH3 in different groups. **I** The relative fluorescence intensities of MPO in different groups. **J** The representative blots of TLR4, MPO, PKC and CitH3 proteins in different groups. **K** The expression levels of TLR4 protein in different groups. **L** The expression levels of PKC protein in different groups. **M** The expression levels of CitH3 protein in different groups. **N** The expression levels of MPO protein in different groups. n = 3, $$\overline{x}$$± *s*, ^#^*P* < 0.05, ^##^*P* < 0.01, and ^###^*P* < 0.001 vs. Con; ^@^*P* < 0.05, ^@@^*P* < 0.01, and ^@@@^*P* < 0.001 vs. AGA; ^$^*P* < 0.05, ^$$^*P* < 0.01, and ^$$$^*P* < 0.001 vs. 2.5 g/kg SGs; ^&^*P* < 0.05, ^&&^*P* < 0.01, and ^&&&^*P* < 0.001 vs. 5 g/kg SGs; ^*^P < 0.05, ^**^P < 0.01, and ^***^P < 0.001 vs. 10 g/kg SGs

To confirm the formation of NETs in the AGA rat model, the markers of NETs, including NE, MPO and CitH3, were examined. The results showed that the AGA model group had significantly higher levels of NE, MPO, and CitH3 in serum compared to the control group (*P* < 0.01). However, treatment with three doses of SGs significantly reduced the serum levels of NE (*P* < 0.05, Fig. 8A**)**, MPO (*P* < 0.001, Fig. 8B), and CitH3 (*P* < 0.001, Fig. 8C) with statistical significance. Moreover, the levels of MPO and the inflammatory factor TNF-α were positively correlated (r^2^ = 0.6 393, *P* < 0.0 001, Fig. 8D). Similarly, the level of CitH3 was positively correlated with the levels of IL-1β (r^2^ = 0.6708, *P* < 0.0001, Fig. 8E) and TNF-α (r^2^ = 0.5892, *P* < 0.0001, Fig. 8F).

To determine the effects of SGs on the formation of NETs in AGA rats, the formation of NETs in the ankle joint was detected by immunofluorescence co-localization. We found that the levels of CitH3, MPO and their co-localization were significantly higher in the AGA model group compared to that in the control group (*P* < 0.001, Fig. 8G). This indicated that NETs were formed in the ankle joints of AGA rats. After SGs were administered, a significant decrease in CitH3 (*P* < 0.001, Fig. 8H) and MPO (*P* < 0.01, Fig. 8I) levels and their co-localization in the ankle joints.

To further investigate the mechanisms of SGs against the formation of NETs, the expression levels of key upstream proteins TLR4 and PKC and the downstream proteins CitH3 and MPO in NETs signaling was determined by Western blotting analysis. The results showed that the expression levels of the TLR4, PKC, CitH3, and MPO proteins were significantly higher in the AGA model group compared to that in the control group (*P* < 0.01). All three doses of SGs significantly decreased the expression level of the TLR4 (*P* < 0.05, Fig. 8J and K), PKC (*P* < 0.05, Fig. 8J and L), CitH3 (*P* < 0.001, Fig. 8J and M) and MPO (*P* < 0.01, Fig. 8J and N) proteins.

## Discussion

Several studies have shown the “immune-inflammation” imbalance and energy metabolism disorder are important pathological events during the progression of AGA [39–41]. Several signal pathways, such as the parathyroid hormone synthesis, secretion and action, the regulation of lipolysis in adipocytes, and the linoleic acid metabolism, were enriched by GSEA, indicating that the material and energy metabolism disorders are involved into the pathological processes of AGA. Additionally, the results of GSEA and functional enrichment analysis also indicated that the inflammatory response, chemotaxis of various neutrophil and inflammatory factors are all activated during the progression of AGA, accompanied by the formation of NETs, suggesting that an imbalance in the “immune-inflammation” system may be a distinguishing feature of AGA. It has been reported that neutrophils are the predominant cells in the joint fluid of patients with AGA [42]. The accumulation of MSU crystals, acting as an endogenous danger signal, triggers a robust infiltration of neutrophils into the affected joint area [43]. This influx activates the body’s innate immune response, leading to the release of various pro-inflammatory cytokines, chemokines, ROS, and other mediators [7, 44]. This cascade of events further enhances the recruitment and activation of neutrophils, ultimately resulting in an increased concentration of neutrophils at the site of inflammation in the joint. Notably, MSU crystals can activate neutrophils, leading to the formation of NETs [45, 46], a fibrous meshwork of histones, cytoplasmic proteins, proteases, and a number of small granular proteins attached to the surface of a DNA-based skeleton, which can play an important role in the immune regulation of the body.

Current studies have reported that NETs have both pro-inflammatory and anti-inflammatory roles in AGA [42, 47–49]. On the one hand, NETs are thought to play an important role in triggering inflammation in the early stages of AGA. The formation of NETs is a hallmark of increased AGA bursts and is a key factor contributing to the pathology of AGA [50]. NETs can cause tissue damage and trigger inflammation in AGA in a variety of ways. Firstly, histones and granulins from NETs are able to activate the complement system and promote the recruitment of other immune cells (e.g. monocytes and macrophages) to the site of inflammation [51]. The recruited immune cells further propagate inflammation by secreting pro-inflammatory cytokines such as IL-1β, TNF-α and IL-6 [52, 53]. Furthermore, NETs can directly promote inflammation through various pro-inflammatory molecules (e.g., histones, DNA, and granulin) which can activate NLRP3 inflammatory vesicles and promote the release of IL-1β [54]. Last but not least, NETs can cause mitochondrial damage, leading to the release of mtDNA, which is recognized by NLRP3 inflammatory vesicles, further exacerbating the inflammatory response [55]. This is the main reason for the manifestation of typical symptoms such as severe pain in the joints, swelling, etc. [56, 57]. On the other hand, the gradual increase in the number of NETs formed with the development of AGA may have led to a large-scale accumulation of NETs, and the high concentration of NETs adhered to form a special type of aggregated mesh structure aggregated NETs (aggNETs). Schauer et al. [47] demonstrated that aggNETs were able to rapidly capture pro-inflammatory cytokines and chemokines through their large number of intrinsic serine proteases on their surfaces and were able to catabolize levels of pro-inflammatory mediators preventing neutrophil aggregation and controlling the exacerbation of the inflammatory response to AGA. However, this process should occur during the period of AGA inflammation subsiding, as Laurent L Reber et al. [48] also demonstrated that aggNETs did not exert an anti-inflammatory effect in an early in vivo model of MSU-induced AGA. In addition, aggNETs were able to tightly wrap around the surface of MSU crystals to isolate them from the surrounding inflammatory mediators, thus relieving inflammation, which is consistent with the observation that the symptoms of AGA can be relieved on their own after 2–3 d of attack, and that the intense inflammatory response gradually subsides. AggNETs are an important mechanism for the inflammatory regression of AGA. Our findings suggested that NETs may strongly influence in the development of AGA, and targeting this pathway might be an effective strategy for treating AGA.

The SGs contained *Clematidis radix et rhizoma, Saposhnikoviae radix and Cyathulae radix*, which are commonly used to treat arthritic diseases such as rheumatoid arthritis and osteoarthritis [58–60]. *Lonice raejaponicae caulis, Forsythiae fructus, Phellodendri chinensis cortex,* etc. have all been reported to reduce the level of inflammation and increase energy metabolism in the body [61–64]. According to the Chinese Pharmacopoeia, SGs are recommended for patients with damp-heat syndrome, which may be characterized by symptoms such as sweating, joint heat, and an increase in the level of inflammatory factors. These symptoms are commonly associated with rheumatic diseases and AGA [65]. Therefore, we investigated whether SGs can be used to treat AGA in clinics, along with its other known therapeutic effects. For that we also constructed the active ingredient-target-pathway network of SGs related to the treatment of AGA and found that SGs might alleviate the severity of the disease by regulating the imbalance between immune response and inflammation, as well as energy metabolism disorder. NETs pathway, which contained 36 core targets of SGs, such as TLR4, SYK, PI3K, PKC, NF-κB, MPO, NE, etc., was well-matched to the efficacy of SGs. NETs might be one of the main pathways by which SGs act on AGA. SGs significantly regulated the expression of key genes in this pathway, including the pattern recognition receptor TLR4 of MSU, the key enzyme PKC in signal transduction, the marker proteins MPO and NE associated with the NETs pathway, and the key protein NF-κB produced by inflammatory factors.

To verify our hypothesis, the MSU-induced AGA rat model was used. During the experiment, the AGA rats exhibited symptoms such as ankle swelling, ankle disability, local heating of the ankle joints, and pain in the ankle joints with prominent inflammatory infiltration and damage to the cartilage in the ankle joints [66]. Further analysis was conducted to detect markers and proteins related to NETs, and the results showed a significant increase in the generation of NETs in the serum and ankle joint lesions of AGA rats. Administering SGs significantly alleviated disease severity, decreased the content of NETs, and reduced the level of expression PKC, CitH3, and MPO. A very strong correlation was found between serum levels of inflammatory factors and markers of NETs, indicating the therapeutic effects of SGs may be associated with the corresponding inhibitory effects in the NETs pathway, which were also in line with our bioinformatics and network analysis.

## Conclusion

To summarize, our findings reveal that SGs may effectively alleviate the severity of AGA by suppressing NETs-promoted imbalance between immunity and inflammation, which not only shed light into broadening the clinical indications of SGs, but benefit AGA therapy utilizing clinical pharmacology in the drug repurposing.

### Supplementary Information

Additional file 1: Table S1. Identification of the components in positive ion mode in SGs. Table S2. Identification of the components in negative ion mode in SGs.

## Data Availability

All data for the duration of the study can be found in this article and appendix.

## Abbreviations

AGA: Acute gouty arthritis
CitH3: Citrullinated histone H3
DEGs: Differential expressed genes
DRG: Dorsal root ganglion
EDTA: Ethylene diamine tetraacetic acid
GO: Gene Ontology
GS: Gene significance
GSEA: Gene set enrichment analysis
IL-1β: Interleukin-1beta
IL-6: Interleukin-6
KEGG: Kyoto Encyclopedia of Genes and Genomes
MM: Module membership
MPO: Myeloperoxidase
MSU: Monosodium urate
NE: Neutrophil elastase
NETs: Neutrophil extracellular traps
PBMC: Peripheral blood mononuclear cells
PPI: Protein–protein interaction
RIPA: Radio immunoprecipitation assay
SGs: Shirebi granules
TNF- α: Tumor necrosis factor alpha
WGCNA: Weighted gene co-expression network analysis
