# Supplementary material for: Shirebi granules ameliorate acute gouty arthritis by inhibiting NETs-induced imbalance between immunity and inflammation
Source: Chin Med. 2024 Aug 9;19:105. doi: 10.1186/s13020-024-00962-6 (PMC11312299; doi:10.1186/s13020-024-00962-6)
Supplement: Supplementary file 1 — Additional file 1: Table S1. Identification of the components in positive ion mode in SGs. Table S2. Identification of the components in negative ion mode in SGs. [file 13020_2024_962_MOESM1_ESM.docx]

**Supplementary Material for Shirebi Granules ameliorate acute gouty arthritis by inhibiting NETs-induced imbalance between immunity and inflammation**

**1.Table S1 Identification of the components in positive ion mode in SGs**

**2.Table S2 Identification of the components in negative ion mode in SGs**

**Table S1 Identification of the components in positive ion mode in SGs**

| Peak no. | Component name | RT (min) | Neutral mass  (Da) | Observed *m/z* | Formula | Mass error (ppm) | Adducts | MS/MS | Source |
| --- | --- | --- | --- | --- | --- | --- | --- | --- | --- |
| 1 | arginine | 0.51 | 174.1118 | 175.1191 | C_6_H_14_N_4_O_2_ | 0.7 | [M+H]^+^ | 104.1094 | *Cyathulae radix* |
| 3 | Maltose | 0.65 | 342.1156 | 343.1239 | C_12_H_22_O_11_ | 1.1 | [M+H]^+^ | 281.07682, 203.05015 | *Mori ramulus* |
| 5 | Clove Oil Glycerin | 0.85 | 244.0949 | 262.1284 | C_11_H_16_O_6_ | -0.3 | [M+NH_4_]^+^ | 127.03992 | *Phellodendri chinensis cortex* |
| 6 | 4-hydroxycinnamic acid | 1.23 | 164.048 | 182.0818 | C_9_H_8_O_3_ | 3.5 | [M+NH_4_]^+^ | 147.04587 | *lonice raejaponicae caulis, Forsythiae fructus* |
| 7 | tyrosine | 1.23 | 181.0745 | 182.0818 | C_9_H_11_NO_3_ | 3.5 | [M+H]^+^ | 165.05662, 147.04587, 136.07707, 91.05385 | *Cyathulae radix* |
| 9 | Aristolochic acid A | 1.43 | 345.0472 | 346.0534 | C_16_H_11_NO_8_ | -2.4 | [M+H]^+^ | 326.1222, 294.1567, 276.1458 | *Stephaniae tetrandrae radix* |
| 10 | Tazopsine | 1.53 | 349.153 | 350.1602 | C_18_H_23_NO_6_ | 1.2 | [M+H]^+^ | 300.12526, 181.06473 | *Stephaniae tetrandrae radix* |
| 11 | adenosine | 1.56 | 267.0973 | 268.1046 | C_10_H_13_N_5_O_4_ | 2.1 | [M+H]^+^ | 136.06255, 119.03488 | *Forsythiae fructus, Pheretima* |
| 12 | Deoxyguanosine | 1.56 | 267.0973 | 268.1046 | C_10_H_13_N_5_O_4_ | 2.1 | [M+H]^+^ | 136.06255 | *Phellodendri chinensis cortex* |
| 14 | Guanosine | 1.72 | 283.0918 | 284.0991 | C_10_H_13_N_5_O_5_ | 0.6 | [M+H]^+^ | 269.08798, 152.05752, 135.03192 | *Phellodendri chinensis cortex* |
| 17 | L-phenylalanine | 2.37 | 165.0794 | 166.0867 | C_9_H_11_NO_2_ | 2.6 | [M+H]^+^ | 120.08129, 103.05413, 91.05336 | *Atractylodis rhizoma* |
| 18 | phenylalanine | 2.37 | 165.0794 | 166.0867 | C_9_H_11_NO_2_ | 2.6 | [M+H]^+^ | 120.08129, 103.05413, 91.05336 | *Cyathulae radix* |
| 19 | trans-Cinnamic acid | 2.37 | 148.0529 | 166.0867 | C_9_H_8_O_2_ | 2.6 | [M+NH_4_]^+^ | 103.05413, 91.05336 | *lonice raejaponicae caulis, Phellodendri chinensis cortex* |
| 25 | 3,3'-Dimethoxy 4,4',9-trihydroxy-7,9'-epoxylignin-7'-one | 4.07 | 374.1367 | 392.1706 | C_20_H_22_O_7_ | 0.5 | [M+NH_4_]^+^ | 137.06018, 122.03944 | *Forsythiae fructus* |
| 26 | Hyuganin D | 4.07 | 374.1367 | 392.1706 | C_20_H_22_O_7_ | 0.5 | [M+NH_4_]^+^ | 137.06018, 122.03944 | *Saposhnikoviae radix* |
| 32 | 4-O-caffeoylquinic acid | 4.63 | 354.0957 | 355.103 | C_16_H_18_O_9_ | 1.8 | [M+H]^+^ | 163.03911, 145.02853 | *Atractylodis rhizoma* |
| 33 | Neochlorogenic acid | 4.63 | 354.0957 | 355.103 | C_16_H_18_O_9_ | 1.8 | [M+H]^+^ | 163.03911, 145.02853 | *lonice raejaponicae caulis* |
| 38 | 3-Methoxy-4-hydroxyphenol-1-O-*α*-L-rhamnose-(1''→6')-*β*-D-glucopyranoside | 5.61 | 448.1601 | 471.1493 | C_19_H_28_O_12_ | 4.3 | [M+Na]^+^ | 479.15632, 325.09195, 163.03978, 145.02974 | *Phellodendri chinensis cortex* |
| 40 | forsythoside E | 6.24 | 462.1715 | 480.2053 | C_20_H_30_O_12_ | -4.7 | [M+NH_4_]^+^ | 317.1223, 300.1591 | *Forsythiae fructus* |
| 47 | suspensaside C | 6.84 | 460.1574 | 483.1501 | C_20_H_28_O_12_ | 2.8 | [M+Na]^+^ | 153.0180, 121.0297 | *Forsythiae fructus* |
| 49 | chlorogenic acid | 6.98 | 354.0957 | 355.103 | C_16_H_18_O_9_ | 1.8 | [M+H]^+^ | 163.03911, 145.02853 | *lonice raejaponicae caulis, Phellodendri chinensis cortex, Forsythiae fructus* |
| 56 | (-)-chorogenic acid methyl ester | 7.45 | 368.1108 | 369.1181 | C_17_H_20_O_9_ | 1.8 | [M+H]^+^ | 145.02989 | *Phellodendri chinensis cortex* |
| 60 | UndulatosideA | 7.51 | 354.0957 | 355.103 | C_16_H_18_O_9_ | 1.8 | [M+H]^+^ | 163.03911, 145.02853 | *Saposhnikoviae radix* |
| 62 | mulberroside A | 7.68 | 568.1764 | 569.1837 | C_26_H_32_O_14_ | -4.9 | [M+H]^+^ | 407.13419, 245.08105, 227.06974, 135.04476 | *Mori ramulus* |
| 63 | clematichinenol | 7.7 | 244.0733 | 245.0806 | C_14_H_12_O_4_ | -1.0 | [M+H]^+^ | 227.06974 | *Clematidis radix et rhizoma* |
| 64 | Cudranin | 7.7 | 244.0733 | 245.0806 | C_14_H_12_O_4_ | -1.0 | [M+H]^+^ | 227.06974, 135.04476 | *Mori ramulus* |
| 65 | Oxyresveratrol 2-O-*β*-D-glucopyranoside | 7.7 | 406.1257 | 407.133 | C_20_H_22_O_9_ | -1.7 | [M+H]^+^ | 245.08105, 227.06974, 135.04476 | *Mori ramulus* |
| 68 | (2E,6S)-8-[*α*-L-arabinopyranosyl (1"→6')-*β*-D-Glucopyranosyloxy]-2,6-dimethyloct-2-eno-1,2"-lactone | 8.33 | 462.2119 | 462.2114 | C_21_H_34_O_11_ | 3.9 | [M-e]^+^ | 300.15840, 107.04871 | *lonice raejaponicae caulis* |
| 72 | moracin C | 8.55 | 310.12 | 328.1538 | C_19_H_18_O_4_ | -1.7 | [M+NH_4_]^+^ | 267.06432, 239.07121 | *Mori ramulus* |
| 73 | (+)-lariciresinol 4,4'-O-bis-*β*-D-glucopyranoside | 9.27 | 358.1263 | 717.2585 | C_32_H_44_O_18_ | -2.1 | [M+H]^+^ | 197.08172, 179.07281, 127.04003 | *lonice raejaponicae caulis, Cyathulae radix* |
| 75 | sweroside | 9.3 | 716.2513 | 359.1336 | C_16_H_22_O_9_ | -0.2 | [M+H]^+^ | 525.15995, 381.11641 | *Clematidis radix et rhizoma* |
| 76 | Butyl 5-O-caffeoylquinate | 9.4 | 410.1518 | 411.159 | C_20_H_26_O_9_ | 2.0 | [M+H]^+^ | 342.1685, 179.0719 | *lonice raejaponicae caulis* |
| 77 | (+)-tembetarine | 9.65 | 344.1854 | 344.1848 | C_20_H_26_NO_4_ | -2.4 | [M-e]^+^ | 272.12631, 145.02872, 137.06185 | *Phellodendri chinensis cortex* |
| 79 | (2S,3S)-3-(4-hydroxy-3-methoxyphenyl)-3-methoxypropane-1,2-diol | 9.88 | 228.1001 | 229.1074 | C_11_H_16_O_5_ | 1.4 | [M+H]^+^ | 211.09466, 197.08299, 179.07135, 151.07622 | *Forsythiae fructus* |
| 80 | 3-(4-hydroxy-3,5-dimethoxyphenyl)-propane-1,2-diol | 9.88 | 228.1001 | 229.1074 | C_11_H_16_O_5_ | 1.4 | [M+H]^+^ | 211.09466, 197.08299, 179.07135, 151.07622 | *Cyathulae radix* |
| 81 | Loganin | 9.88 | 390.1524 | 413.1416 | C_17_H_26_O_10_ | -0.5 | [M+Na]^+^ | 341.12421, 229.10722, 197.08299, 179.0713 | *lonice raejaponicae caulis* |
| 82 | loganin aglycone | 9.88 | 228.1001 | 229.1074 | C_11_H_16_O_5_ | 1.4 | [M+H]^+^ | 197.08299, 179.07135, 151.07622 | *lonice raejaponicae caulis* |
| 84 | (+)-fuzitine | 10.42 | 342.1675 | 342.1694 | C_20_H_24_NO_4_ | 1.7 | [M-e]^+^ | 297.1136, 282.0892, 265.0858, 191.0862 | *Phellodendri chinensis cortex* |
| 85 | 5-O-feruloylquinic acid | 10.68 | 368.1108 | 369.1181 | C_17_H_20_O_9_ | 0.1 | [M+H]^+^ | 177.05627, 145.02989 | *Atractylodis rhizoma, Phellodendri chinensis cortex* |
| 86 | Methyl 5-O-caffeoylquinate | 10.69 | 368.1108 | 369.1181 | C_17_H_20_O_9_ | 0.1 | [M+H]^+^ | 145.02989 | *lonice raejaponicae caulis* |
| 87 | 3-hydroxy-3-methyl-5-oxo-5-(3,4,5-trihydroxy-6-((7-hydroxy-1-(4-hydroxybenzyl)-2-methyl-1,2,3,4tetrahydroisoquinolin-8-yl)oxy)tetrahydro-2H-pyran-2-yl)methoxy)pentanoic acid | 10.82 | 591.2324 | 592.2397 | C_29_H_37_NO_12_ | 1.5 | [M+H]^+^ | 530.24373, 286.14397, 255.10167, 163.04001 | *Phellodendri chinensis cortex* |
| 89 | (−)-oblongine | 11.23 | 314.1752 | 314.1747 | C_19_H_24_NO_3_ | -1.2 | [M-e]^+^ | 269.11633, 239.06938, 107.04983 | *Phellodendri chinensis cortex* |
| 92 | icariside D1[phenethyl-*β*-D-apiopyranosyl(1→6)-*β*-D-glucopyranoside] | 11.35 | 416.1677 | 439.1587 | C_19_H_28_O_10_ | 2.8 | [M+Na]^+^ | 329.10219, 151.03968, 125.02469 | *Atractylodis rhizoma* |
| 94 | 7-O-butylsecologanic acid | 12.13 | 430.1859 | 453.1752 | C_20_H_30_O_10_ | 4.5 | [M+Na]^+^ | 203.07063 | *lonice raejaponicae caulis* |
| 95 | japenol | 12.26 | 376.1518 | 399.141 | C_20_H_24_O_7_ | -0.4 | [M+Na]^+^ | 203.03433, 179.03472, 161.02445, 151.03969 | *lonice raejaponicae caulis* |
| 98 | (7E)-sinapate-4-O-*β*-D-glucopyranoside | 12.53 | 386.1209 | 409.11 | C_17_H_22_O_10_ | -1.3 | [M+Na]^+^ | 203.03487, 179.03480, 161.02430, 133.02896 | *Atractylodis rhizoma* |
| 100 | 4-Hydroxy-3,5-dimethoxycinnamic acid | 12.58 | 224.0781 | 207.0665 | C_11_H_12_O_5_ | -0.5 | [M+H-H2O]^+^ | 192.0684, 164.0739 | *Coicis semen* |
| 101 | Prim-O-glucosylcimifugin | 12.76 | 468.1627 | 469.17 | C_22_H_28_O_11_ | -1.0 | [M+H]^+^ | 343.1785, 356.1867, 307.1179 | *Saposhnikoviae radix* |
| 102 | Isotrilobine-N-2-oxide | 12.78 | 592.2596 | 631.2248 | C_36_H_36_N_2_O_6_ | 4.3 | [M+K]^+^ | 359.11242 | *Stephaniae tetrandrae radix* |
| 103 | (+)-pinoresinol 4-O-*β*-D-glucopyranoside | 13.22 | 520.1938 | 538.2276 | C_26_H_32_O_11_ | -1.3 | [M+NH_4_]^+^ | 298.14805, 146.02542 | *Clematidis radix et rhizoma* |
| 104 | L-phenylalaninosecologanin | 13.22 | 537.2203 | 538.2276 | C_26_H_35_NO_11_ | -1.3 | [M+H]^+^ | 469.17209, 358.16045, 298.14805, 256.12954, 146.02542 | *lonice raejaponicae caulis* |
| 106 | corypalmine | 13.23 | 341.1622 | 342.1695 | C_20_H_23_NO_4_ | -1.4 | [M+H]^+^ | 312.12390, 192.10248, 177.07909, 148.07649 | *Phellodendri chinensis cortex* |
| 107 | Cyclanoline | 13.25 | 342.17 | 342.1695 | C_20_H_24_NO_4_ | -1.4 | [M-e]^+^ | 312.12390, 192.10248, 177.07909 | *Stephaniae tetrandrae radix* |
| 108 | (+)-8-hydroxyepipinores inol-4-O-*β*-D-glucopyranoside | 13.64 | 536.1893 | 559.1806 | C_26_H_32_O_12_ | 2.0 | [M+Na]^+^ | 328.09381 | *Forsythiae fructus* |
| 111 | glochidioboside | 13.75 | 522.2104 | 545.2016 | C_26_H_34_O_11_ | 4.1 | [M+Na]^+^ | 359.14843, 344.12557, 313.10803, 241.04963, 189.05430 | *Forsythiae fructus* |
| 112 | Isoforsythiaside | 14.19 | 624.2048 | 625.2121 | C_29_H_36_O_15_ | -1.0 | [M+H]^+^ | 479.15632, 325.09195, 163.03978, 145.02974 | *Forsythiae fructus* |
| 113 | (+)-isolariciresinol-6*α*-O-*β*-D-glucopyranoside | 14.43 | 522.2099 | 545.2018 | C_26_H_34_O_11_ | 2.5 | [M+Na]^+^ | 329.13882, 293.04469, 123.04429 | *Forsythiae fructus* |
| 116 | morin | 14.7 | 302.0424 | 303.0497 | C_15_H_10_O_7_ | -0.9 | [M+H]^+^ | 163.03941, 145.03205 | *Mori ramulus* |
| 117 | Quercetin | 14.7 | 302.0424 | 303.0497 | C_15_H_10_O_7_ | -0.9 | [M+H]^+^ | 163.03941, 145.03205 | *lonice raejaponicae caulis, Phellodendri chinensis cortex, Forsythiae fructus* |
| 119 | 4-hydroxy-3-methoxyphenol-*β*-D-apiopyranosyl(1→6)-D-glucopyranoside | 14.79 | 434.1443 | 457.1336 | C_18_H_26_O_12_ | 1.9 | [M+Na]^+^ | 163.03941, 145.03205 | *Atractylodis rhizoma* |
| 120 | Canthoside C | 14.79 | 434.1443 | 457.1336 | C_18_H_26_O_12_ | 1.9 | [M+Na]^+^ | 163.03941, 145.03205 | *Phellodendri chinensis cortex* |
| 121 | Matrine C | 14.79 | 434.1443 | 457.1336 | C_18_H_26_O_12_ | 1.9 | [M+Na]^+^ | 163.03941, 145.03205 | *Phellodendri chinensis cortex* |
| 122 | rutin | 14.8 | 610.1511 | 611.1626 | C_27_H_30_O_16_ | 1.9 | [M+H]^+^ | 301.03331 | *lonice raejaponicae caulis, Coicis semen, Forsythiae fructus* |
| 123 | (+)-lyoniresinol 9'-O-*β*-D-glucopyranoside | 15.28 | 582.2306 | 605.2235 | C_28_H_38_O_13_ | 3.0 | [M+Na]^+^ | 389.15981, 359.14860, 208.07349, 165.05497 | *Clematidis radix et rhizoma* |
| 125 | (+)-menisperine | 15.53 | 356.1853 | 356.1848 | C_21_H_26_NO_4_ | -0.8 | [M-e]^+^ | 192.10187, 177.08061 | *Phellodendri chinensis cortex* |
| 126 | forsythoside A | 15.57 | 624.2035 | 642.2373 | C_29_H_36_O_15_ | -3.0 | [M+NH_4_]^+^ | 472.1519, 325.0922 | *Forsythiae fructus* |
| 127 | forsythoside B | 15.71 | 756.2479 | 779.2446 | C_34_H_44_O_19_ | 3.9 | [M+Na]^+^ | 623, 593.20855, 447.15067, 315.10812, 161 | *Forsythiae fructus* |
| 128 | forsythoside I | 15.92 | 624.2052 | 647.1944 | C_29_H_36_O_15_ | -0.4 | [M+Na]^+^ | 471.14870, 325.09210, 309.09670, 163.03994, 145.02858 | *Forsythiae fructus* |
| 129 | cimifugin | 16.1 | 306.1106 | 307.1178 | C_16_H_18_O_6_ | 0.8 | [M+H]^+^ | 259.0594, 235.0610 | *Saposhnikoviae radix* |
| 130 | Plantainoside A | 16.16 | 478.1471 | 479.1544 | C_23_H_26_O_11_ | -0.8 | [M+H]^+^ | 325.09210, 309.09670, 163.03994, 145.02858 | *Forsythiae fructus* |
| 131 | berlambine | 16.2 | 351.1104 | 352.1177 | C_20_H_17_NO_5_ | -0.7 | [M+H]^+^ | 336.08311, 308.09049, 294.07818 | *Phellodendri chinensis cortex* |
| 132 | tetrahydroxystilbene | 16.54 | 244.07356 | 245.1383 | C_14_H_12_O_4_ | 5.0 | [M+H]^+^ | 227.1305, 192.1024 | *Mori ramulus* |
| 133 | matairesinoside | 16.75 | 520.1953 | 543.1846 | C_26_H_32_O_11_ | 1.6 | [M+Na]^+^ | 235.09880, 175.07728, 137.05984 | *Forsythiae fructus* |
| 137 | calceolarioside A | 16.96 | 478.147 | 479.1548 | C_22_H_24_O_9_ | 0.1 | [M+H]^+^ | 193.05093, 121.02916 | *Clematidis radix et rhizoma* |
| 141 | rhaponterone | 17.49 | 420.1436 | 420.1431 | C_21_H_24_O_9_ | 3.8 | [M-e]^+^ | 278.08307 | *Cyathulae radix* |
| 142 | 5-O-Methylvisammioside | 17.84 | 452.1679 | 453.1752 | C_22_H_28_O_10_ | -0.8 | [M+H]^+^ | 420.1449, 305.1526, 243.0638 | *Saposhnikoviae radix* |
| 143 | icariside F2[benzyl-*β*-D-apiopyranosyl(1→6)-*β*-D-glucopyranoside] | 17.84 | 430.1859 | 453.1752 | C_20_H_30_O_10_ | 4.5 | [M+Na]^+^ | 191.07085 | *Atractylodis rhizoma* |
| 144 | Fangchinoline | 17.92 | 608.2882 | 609.2954 | C_37_H_40_N_2_O_6_ | -0.8 | [M+H]^+^ | 367.16378, 192.10402 | *Stephaniae tetrandrae radix* |
| 145 | 3,4-dihydroxy-allylbenzene | 18.35 | 150.06808 | 151.0771 | C_9_H_10_O_2_ | 1.7 | [M+H]^+^ | 133.0695, 105.0719 | *Forsythiae fructus* |
| 150 | 25S-inokosterone | 19.02 | 480.3103 | 503.2995 | C_27_H_44_O_7_ | 3.2 | [M+Na]^+^ | 411.25060, 329.21074, 301.17905, 283.17111, 173.09703 | *Cyathulae radix* |
| 151 | cyasterone | 19.02 | 520.3031 | 521.3104 | C_29_H_44_O_8_ | -1.0 | [M+H]^+^ | 485.2924, 329.21074, 301.17905, 283.17111, 173.09703, 131.08440 | *Cyathulae radix* |
| 152 | stachysterone D | 19.02 | 462.2987 | 485.2879 | C_27_H_42_O_6_ | 1.2 | [M+Na]^+^ | 411.25060, 329.21074, 301.17905, 283.17111, 225.12674, 173.09703, 109.06462 | *Cyathulae radix* |
| 153 | 7'-epi-8-hydroxypinoresinol | 19.15 | 374.1367 | 392.1706 | C_20_H_22_O_7_ | 0.5 | [M+NH_4_]^+^ | 137.06018, 122.03944 | *Forsythiae fructus* |
| 154 | Resveratrol | 19.15 | 228.0794 | 229.0957 | C_14_H_12_O_3_ | 4.3 | [M+H]^+^ | 211.9078 | *Mori ramulus* |
| 156 | clemaphenol A | 19.26 | 358.141 | 359.1483 | C_20_H_22_O_6_ | -1.8 | [M+H]^+^ | 137.06018, 122.03944 | *Clematidis radix et rhizoma* |
| 157 | (+)-epipinoresinol-4'-O-*β*-D-glucopyranoside | 19.28 | 520.1953 | 543.1846 | C_26_H_32_O_11_ | 1.6 | [M+Na]^+^ | 235.09880, 175.07728 | *Forsythiae fructus* |
| 159 | columbamine | 19.31 | 338.1393 | 338.1388 | C_20_H_20_NO_4_ | 0.3 | [M-e]^+^ | 322.10465, 307.08607, 279.08935 | *Phellodendri chinensis cortex* |
| 160 | Dehydrocrebanine | 19.31 | 337.1315 | 338.1388 | C_20_H_19_NO_4_ | 0.3 | [M+H]^+^ | 322.10465, 279.08935 | *Stephaniae tetrandrae radix* |
| 161 | grandifloroside | 19.69 | 538.1687 | 561.1606 | C_25_H_30_O_13_ | 2.7 | [M+Na]^+^ | 479.15907, 375.12908, 201.01647, 161.02462, 135.04512, 121.02882 | *lonice raejaponicae caulis* |
| 163 | Fenfangjine K | 20.07 | 622.3048 | 623.3121 | C_38_H_42_N_2_O_6_ | 0.9 | [M+H]^+^ | 580.26818, 534.22991, 381.18066, 174.09360 | *Stephaniae tetrandrae radix* |
| 164 | sec-o-glucosylhamaudol | 21.08 | 438.152 | 438.1548 | C_21_H_26_O_10_ | 2.8 | [M-e]^+^ | 437.1448, 312.1240, 275.0939 | *Saposhnikoviae radix* |
| 166 | Ethyl caffeate | 21.83 | 208.0742 | 209.0823 | C_11_H_12_O_4_ | 1.4 | [M+H]^+^ | 118.0646 | *Phellodendri chinensis cortex\|\|lonice raejaponicae caulis* |
| 167 | (+)-1-hydroxypinoresinol-4''-O-*β*-D-glucopyranosi-de | 22.03 | 534.2103 | 557.1999 | C_27_H_34_O_11_ | 0.5 | [M+Na]^+^ | 356.12590, 121.02880 | *Forsythiae fructus* |
| 168 | syringaresinol-4'-O-*β*-D-glucopyranoside | 22.03 | 580.2157 | 603.2079 | C_28_H_36_O_13_ | 3.1 | [M+Na]^+^ | 371.14929, 356.12590, 121.02880 | *Atractylodis rhizoma* |
| 169 | Fenfangjine H | 22.18 | 305.0683 | 306.0756 | C_18_H_11_NO_4_ | -1.6 | [M+H]^+^ | 278.08098, 248.07082 | *Stephaniae tetrandrae radix* |
| 170 | palmarumycin CP2 | 22.18 | 318.089 | 336.1228 | C_20_H_14_O_4_ | -0.7 | [M+NH_4_]^+^ | 278.08098, 248.07082 | *lonice raejaponicae caulis* |
| 171 | (+)-pinoresinol monomethyl ether-4-O-*β*-D-glucoside | 22.19 | 534.2096 | 557.1989 | C_27_H_34_O_11_ | -0.8 | [M+Na]^+^ | 355.15246, 189.09117 | *Forsythiae fructus* |
| 173 | cnidimoide A | 22.65 | 438.1524 | 439.1597 | C_21_H_26_O_10_ | -0.4 | [M+H]^+^ | 336.1215, 277.1088 | *Saposhnikoviae radix* |
| 174 | 4'-O-*β*-D-glucopyranosylvisamminol | 22.66 | 438.1524 | 439.1597 | C_21_H_26_O_10_ | -0.4 | [M+H]^+^ | 277.1088, 259.0957 | *Saposhnikoviae radix* |
| 175 | morachalcone A | 22.68 | 340.1308 | 341.1381 | C_20_H_20_O_5_ | -0.8 | [M+H]^+^ | 137.06103 | *Mori ramulus* |
| 177 | canthin-6-one | 23.01 | 220.0641 | 221.0713 | C_14_H_8_N_2_O | 1.8 | [M+H]^+^ | 167.06225 | *Phellodendri chinensis cortex* |
| 179 | sarsasaponin | 23.32 | 1048.5444 | 1048.5535 | C_51_H_84_O_22_ | 4.6 | [M-e]^+^ | 901.47948, 883.46902, 755.42155, 593.36864, | *Cyathulae radix* |
| 180 | kihadanin B | 23.33 | 486.1909 | 486.1904 | C_26_H_30_O_9_ | 4.0 | [M-e]^+^ | 336.12212, 320.09101, 129.05544 | *Phellodendri chinensis cortex* |
| 181 | 6'-O-*β*-D-glucosylgentiopicroside | 23.51 | 432.1057 | 433.111 | C_21_H_20_O_10_ | -1.9 | [M+H]^+^ | 367.1200 | *Cyathulae radix* |
| 182 | Isorhamnetin-3-O-*β*-D-Glucoside | 23.51 | 478.1109 | 479.1182 | C_22_H_22_O_12_ | -0.4 | [M+H]^+^ | 317.06579 | *lonice raejaponicae caulis* |
| 183 | naringenin | 23.51 | 272.0688 | 273.0781 | C_15_H_12_O_5_ | 0.1 | [M+H]^+^ | 135.0454 | *Clematidis radix et rhizoma* |
| 184 | (+)-pinoresinol | 23.86 | 358.141 | 359.1483 | C_20_H_22_O_6_ | -1.8 | [M+H]^+^ | 137.06018, 122.03944 | *lonice raejaponicae caulis, Clematidis radix et rhizoma, Forsythiae fructus* |
| 188 | huzhangoside B | 24.24 | 1336.6617 | 1337.669 | C_64_H_104_O_29_ | -3.4 | [M+H]^+^ | 925.44407, 353.22921 | *Clematidis radix et rhizoma, Pheretima* |
| 190 | 11,12,15-Trihydroxy-13-en-oc-tadecenoic acid | 24.92 | 330.241 | 353.2302 | C_18_H_34_O_5_ | 1.1 | [M+Na]^+^ | 213.15043 | *lonice raejaponicae caulis* |
| 191 | saponin CP8 | 24.94 | 1016.5208 | 1017.5277 | C_50_H_80_O_21_ | 1.2 | [M+H]^+^ | 737.41294 | *Clematidis radix et rhizoma* |
| 192 | isopimpinellin | 25.03 | 246.054 | 247.0613 | C_13_H_10_O_5_ | 3.2 | [M+H]^+^ | 161.1078, 147.0923 | *Saposhnikoviae radix* |
| 193 | pinelliae palmatum L | 25.04 | 330.241 | 353.2302 | C_18_H_34_O_5_ | 1.1 | [M+Na]^+^ | 213.15043, 195.13803 | *Cyathulae radix* |
| 194 | saponins CP7 | 25.1 | 1000.5248 | 1000.5206 | C_50_H_80_O_20_ | -3.1 | [M-e]^+^ | 899.46467, 883.47055, 737.41215, 575.35915, 179.05622 | *Clematidis radix et rhizoma* |
| 197 | rhamnetin | 25.38 | 316.058 | 317.0628 | C_16_H_12_O_7_ | -2.8 | [M+H]^+^ | 299.01897, 165.01880 | *Forsythiae fructus* |
| 199 | Loniceracetalide A | 25.61 | 460.1951 | 460.1979 | C_21_H_32_O_11_ | 4.0 | [M-e]^+^ | 109.029 | *lonice raejaponicae caulis* |
| 200 | Loniceroside A | 25.64 | 1044.5506 | 1045.5651 | C_52_H_84_O_21_ | 2.3 | [M+H]^+^ | 1029.52814, 897.48557, 883.47076, 865.46264, 733.45295 | *lonice raejaponicae caulis* |
| 203 | Phellodendron acid | 25.87 | 470.1949 | 471.2022 | C_26_H_30_O_8_ | 1.7 | [M+H]^+^ | 161.06102 | *Phellodendri chinensis cortex* |
| 204 | 7*α*,10*α*-epoxyguaiane-4*α*,11-diol | 25.98 | 270.2189 | 293.1975 | C_16_H_30_O_3_ | -0.7 | [M+Na]^+^ | 119.04891 | *Atractylodis rhizoma* |
| 205 | Isobutylparaben | 26.28 | 194.0948 | 195.0921 | C_11_H_14_O_3_ | 3.7 | [M+H]^+^ | 177.1252 | *Forsythiae fructus* |
| 207 | 11(Z)-hexadecenoic Acid | 26.99 | 254.2242 | 277.2128 | C_16_H_30_O_2_ | -1.0 | [M+Na]^+^ | 125.09789 | *Clematidis radix et rhizoma* |
| 209 | mulberrin | 27.08 | 422.172 | 423.1793 | C_25_H_26_O_6_ | -2.3 | [M+H]^+^ | 311.05468, 241.04931 | *Mori ramulus* |
| 210 | 25R-inokosterone | 27.56 | 480.3086 | 503.2992 | C_27_H_44_O_7_ | 1.3 | [M+Na]^+^ | 255.23261 | *Cyathulae radix* |
| 211 | ecdysterone | 27.56 | 480.3086 | 519.262 | C_27_H_44_O_7_ | -4.8 | [M+K]^+^ | 255.23261 | *Cyathulae radix* |
| 212 | amarasterone A | 27.69 | 522.3557 | 522.3551 | C_30_H_50_O_7_ | 0.0 | [M-e]^+^ | 449.28567 | *Cyathulae radix* |
| 213 | hamaudol | 27.9 | 276.1004 | 277.1076 | C_15_H_16_O_5_ | 1.4 | [M+H]^+^ | 262.1441 | *Stephaniae tetrandrae radix* |
| 214 | Dibutyl phthalate | 27.92 | 278.1522 | 301.1414 | C_16_H_22_O_4_ | 1.4 | [M+Na]^+^ | 149.02428, 121.02947 | *lonice raejaponicae caulis, Cyathulae radix* |
| 215 | Phthaloyl Butylene Diester | 27.92 | 278.1522 | 301.1414 | C_16_H_22_O_4_ | 1.4 | [M+Na]^+^ | 149.02428, 121.02947 | *Forsythiae fructus* |
| 216 | phthalic acid,butyl dodecyl ester | 29.68 | 390.2769 | 413.2661 | C_24_H_38_O_4_ | -0.3 | [M+Na]^+^ | 149.02276 | *Mori ramulus* |

**Table S2 Identification of the components in negative ion mode in SGs**

| Peak no. | Component name | RT (min) | Neutral mass   (Da) | Observed *m/z* | Formula | Mass error (ppm) | Adducts | MS/MS | Source |
| --- | --- | --- | --- | --- | --- | --- | --- | --- | --- |
| 1 | arginine | 0.51 | 174.1118 | 173.1054 | C_6_H_14_N_4_O_2_ | 1.8 | [M-H]^-^ | 131.0808 | *Cyathulae radix* |
| 2 | *β*-D-Fructopyranose | 0.55 | 180.0632 | 215.0326 | C_6_H_12_O_6_ | -0.8 | [M+Cl]^-^ | 165.0406, 146.0468 | *Mori ramulus* |
| 3 | Maltose | 0.65 | 342.1156 | 387.1144 | C_12_H_22_O_11_ | -0.1 | [M+HCOO]^-^ | 281.07682, 203.05015 | *Mori ramulus* |
| 4 | D(+)-Raffinose pentahydrate | 0.84 | 504.1689 | 549.1671 | C_18_H_32_O_16_ | -1.4 | [M+HCOO]^-^ | 425.12981, 404.10419, 341.10847, 179.05602, 161.04548 | *Mori ramulus* |
| 7 | tyrosine | 1.23 | 181.0745 | 180.0663 | C_9_H_11_NO_3_ | 0.7 | [M-H]^-^ | 147.0306 | *Cyathulae radix* |
| 8 | Uridine | 1.27 | 244.069 | 243.0617 | C_9_H_12_N_2_O_6_ | -2.2 | [M-H]^-^ | 200.05615, 191.01933 | *lonice raejaponicae caulis, Pheretima* |
| 9 | Aristolochic acid A | 1.43 | 345.0472 | 344.0399 | C_16_H_11_NO_8_ | 0.7 | [M-H]^-^ | 233.01354 | *Stephaniae tetrandrae radix* |
| 10 | Tazopsine | 1.53 | 349.153 | 348.1452 | C_18_H_23_NO_6_ | 3.8 | [M-H]^-^ | 298.1007, 188.0926 | *Stephaniae tetrandrae radix* |
| 13 | *β*-D-Glucoside-1-hydroxy-6-methoxyphenyl | 1.63 | 302.0999 | 361.1135 | C_13_H_18_O_8_ | -1.4 | [M+CH_3_COO]^-^ | 329.08652 | *Phellodendri chinensis cortex* |
| 14 | Guanosine | 1.72 | 283.0918 | 282.0839 | C_10_H_13_N_5_O_5_ | -1.6 | [M-H]^-^ | 269.08798, 152.05752, 135.03192 | *Phellodendri chinensis cortex, Pheretima* |
| 15 | seguinosideB,4-hydroxyphenyl1-O-*β*-D-apiopyranosyl-(1→6)-*β*-D-glucopyranoside | 1.84 | 302.0997 | 361.1135 | C_13_H_18_O_8_ | -1.4 | [M+CH_3_COO]^-^ | 270.10462, 108.02121 | *Atractylodis rhizoma* |
| 16 | tachioside | 1.84 | 302.0997 | 361.1135 | C_13_H_18_O_8_ | -1.4 | [M+CH_3_COO]^-^ | 270.10462, 108.02121 | *lonice raejaponicae caulis* |
| 17 | L-phenylalanine | 2.37 | 165.0794 | 164.0716 | C_9_H_11_NO_2_ | 4.6 | [M-H]^-^ | 147.0455 | *Atractylodis rhizoma* |
| 19 | trans-Cinnamic acid | 2.37 | 148.0529 | 183.0294 | C_9_H_8_O_2_ | 1.2 | [M+Cl]- | 116.9279, 96.9591 | *lonice raejaponicae caulis, Phellodendri chinensis cortex* |
| 20 | rengynic acid-1'-O-*β*-D-glucoside | 2.52 | 336.1418 | 335.1345 | C_14_H_24_O_9_ | -0.8 | [M-H]^-^ | 164.07151 | *Forsythiae fructus* |
| 21 | 3,4,5-Trimethoxyphenyl-1-O-*β*-D-apiose(1→6)-*β*-D-glucopyranoside | 3.12 | 478.1687 | 477.1614 | C_20_H_30_O_13_ | 0.1 | [M-H]^-^ | 151.0405 | *Mori ramulus* |
| 22 | 2-Hydroxybenzoic acid-5-O-*β*-D-glucoside | 3.2 | 316.079 | 315.0717 | C_13_H_16_O_9_ | -1.5 | [M-H]^-^ | 152.01119, 108.02104 | *Phellodendri chinensis cortex* |
| 23 | 2-(3,4-Dihydroxyphenyl)-ethyl-*β*-D-glucoside | 3.94 | 316.1156 | 315.1084 | C_14_H_20_O_8_ | -0.6 | [M-H]^-^ | 134.04712, 123.04496 | *Forsythiae fructus* |
| 24 | cornoside | 3.94 | 316.1156 | 361.1135 | C_14_H_20_O_8_ | -1.4 | [M+HCOO]^-^ | 134.04712, 123.04496 | *Forsythiae fructus* |
| 27 | 3-hydroxy-3,5-dimethoxyphenyl-*β*-D-glucopyranoside | 4.48 | 332.1105 | 331.1032 | C_14_H_20_O_9_ | -0.8 | [M-H]^-^ | 299.07678, 167.03362, 137.02410 | *Phellodendri chinensis cortex* |
| 28 | 4-hydroxy-3-methoxyphenol*β*-D-glucopyranoside | 4.48 | 272.0893 | 331.1032 | C_12_H_16_O_7_ | -0.8 | [M+CH_3_COO]^-^ | 299.07678, 167.03362, 137.02410 | *Atractylodis rhizoma* |
| 29 | arbutin | 4.48 | 272.0893 | 331.1032 | C_12_H_16_O_7_ | -0.8 | [M+CH_3_COO]^-^ | 299.07678, 167.03362, 137.02410 | *Forsythiae fructus* |
| 30 | Loganin acid | 4.48 | 272.0893 | 331.1032 | C_12_H_16_O_7_ | -0.8 | [M+CH_3_COO]^-^ | 299.07678, 167.03362, 137.02410 | *lonice raejaponicae caulis* |
| 31 | adoxosidic acid | 4.6 | 376.1367 | 375.1294 | C_16_H_24_O_10_ | -0.7 | [M-H]^-^ | 213.07659 | *Forsythiae fructus* |
| 32 | 4-O-caffeoylquinic acid | 4.63 | 354.0957 | 353.0876 | C_16_H_18_O_9_ | -0.7 | [M-H]^-^ | 163.03911, 145.02853 | *Atractylodis rhizoma* |
| 33 | Neochlorogenic acid | 4.63 | 354.0957 | 353.0876 | C_16_H_18_O_9_ | -0.7 | [M-H]^-^ | 163.03911, 145.02853 | *lonice raejaponicae caulis* |
| 34 | 3,5-Dimethoxybenzoic acid-4-O-*β*-D-glucoside | 4.72 | 360.1052 | 359.0979 | C_15_H_20_O_10_ | -1.4 | [M-H]^-^ | 197.04534, 182.02174, 138.03169, 123.00835, 95.01255 | *Phellodendri chinensis cortex* |
| 35 | Secologanosid | 4.72 | 314.0997 | 359.0979 | C_14_H_18_O_8_ | -1.4 | [M+HCOO]^-^ | 197.04534, 182.02174, 138.03169, 123.00835, 95.01255 | *lonice raejaponicae caulis* |
| 36 | forsythide | 4.89 | 390.1158 | 389.1085 | C_16_H_22_O_11_ | -1.0 | [M-H]^-^ | 193.05132, 178.02624, 134.03710 | *Forsythiae fructus* |
| 37 | Coumarin-7-O-*α*-L-rhamnose-(1→6)-O-*β*-D-glucopyranoside | 5 | 456.1269 | 515.1407 | C_20_H_24_O_12_ | 0.2 | [M+CH_3_COO]^-^ | 93.03375 | *Mori ramulus* |
| 38 | 3-Methoxy-4-hydroxyphenol-1-O-*α*-L-rhamnose-(1''→6')-*β*-D-glucopyranoside | 5.61 | 448.1601 | 447.1499 | C_19_H_28_O_12_ | -2.1 | [M-H]^-^ | 479.15632, 325.09195, 163.03978, 145.02974 | *Phellodendri chinensis cortex* |
| 39 | Benzoicacid,4-[(*β*-D-glucopyranosyloxy)methyl]-methyl ester | 5.75 | 328.1148 | 373.113 | C_15_H_20_O_8_ | -2.6 | [M+HCOO]^-^ | 149.05945 | *Phellodendri chinensis cortex* |
| 40 | forsythoside E | 6.24 | 462.1715 | 461.1668 | C_20_H_30_O_12_ | 0.8 | [M-H]^-^ | 387.0950, 293.1240 | *Forsythiae fructus* |
| 41 | 2-methoxy-3,4,5-trihydroxy Phenylethanol glycosides | 6.32 | 362.1211 | 421.135 | C_15_H_22_O_10_ | -0.4 | [M+CH_3_COO]^-^ | 179.03520, 113.02404 | *Forsythiae fructus* |
| 42 | (–)-(7R,8S)-Guaiacylglycerol8-O-*β*-D-glucopyranoside | 6.34 | 376.1369 | 375.1297 | C_16_H_24_O_10_ | 0.0 | [M-H]^-^ | 213.07659, 179.03520, 151.07606, 133.02896 | *Phellodendri chinensis cortex* |
| 43 | loganic acid | 6.34 | 376.1369 | 375.1297 | C_16_H_24_O_10_ | 0.0 | [M-H]^-^ | 213.07659, 169.08699, 151.07606, 133.02896, 113.02404, 95.04937 | *lonice raejaponicae caulis* |
| 44 | coniferin | 6.44 | 342.1315 | 387.1282 | C_16_H_22_O_8_ | -3.7 | [M+HCOO]^-^ | 301.0913, 162.0562 | *Phellodendri chinensis cortex\|\|lonice raejaponicae caulis* |
| 45 | 4-hydroxy-3-methoxyphenyl-*β*-xylopyranosyl(1→6)-*β*-glucopyranoside | 6.53 | 434.1421 | 493.156 | C_18_H_26_O_12_ | -0.6 | [M+CH_3_COO]^-^ | 356.14707 | *Atractylodis rhizoma* |
| 46 | syringin | 6.71 | 372.142 | 431.1559 | C_17_H_24_O_9_ | 0.0 | [M+CH_3_COO]^-^ | 177.01801, 134.03614 | *Atractylodis rhizoma, Cyathulae radix, Phellodendri chinensis cortex* |
| 47 | suspensaside C | 6.84 | 460.1574 | 459.1501 | C_20_H_28_O_12_ | -1.5 | [M-H]^-^ | 151.03994, 123.04451 | *Forsythiae fructus* |
| 48 | adoxosidic acid isomer | 6.85 | 376.1365 | 375.1292 | C_16_H_24_O_10_ | -1.2 | [M-H]^-^ | 195.06562 | *Forsythiae fructus* |
| 49 | chlorogenic acid | 6.98 | 354.0957 | 353.0873 | C_16_H_18_O_9_ | -1.6 | [M-H]^-^ | 163.03911, 145.02853 | *Atractylodis rhizoma, Phellodendri chinensis cortex, Forsythiae fructus* |
| 50 | quinic acid | 6.98 | 192.0631 | 191.0558 | C_7_H_12_O_6_ | -1.7 | [M-H]^-^ | 93.03317 | *Forsythiae fructus* |
| 51 | 3-Hydroxy-4-methoxycinnamic acid | 7.02 | 194.0574 | 239.0556 | C_10_H_10_O_4_ | -2.3 | [M+HCOO]^-^ | 179.03456, 161.02396, 149.06071 | *Clematidis radix et rhizoma* |
| 52 | Ferulic Acid | 7.02 | 194.0574 | 239.0556 | C_10_H_10_O_4_ | -2.3 | [M+HCOO]^-^ | 179.03456, 161.02396, 149.06071 | *lonice raejaponicae caulis, Phellodendri chinensis cortex, Coicis semen* |
| 53 | trans-ferulic acid | 7.02 | 194.0574 | 239.0556 | C_10_H_10_O_4_ | -2.3 | [M+HCOO]^-^ | 179.03456, 161.02396, 149.06071 | *Forsythiae fructus* |
| 54 | caffeic acid | 7.12 | 180.0421 | 179.0348 | C_9_H_8_O_4_ | -0.9 | [M-H]^-^ | 135.04485 | *Coicis semen, Cyathulae radix, lonice raejaponicae caulis, Phellodendri chinensis cortex, Forsythiae fructus* |
| 55 | Secologanoside-7-methylester | 7.28 | 390.1157 | 389.1084 | C_16_H_22_O_11_ | -1.3 | [M-H]^-^ | 329.05089, 165.05619, 121.02909 | *lonice raejaponicae caulis* |
| 56 | (-)-chorogenic acid methyl ester | 7.45 | 368.1108 | 367.103 | C_17_H_20_O_9_ | -1.1 | [M-H]^-^ | 145.02989 | *Phellodendri chinensis cortex* |
| 57 | chlorogenic acid methyl ester | 7.45 | 368.1103 | 367.103 | C_17_H_20_O_9_ | -1.1 | [M-H]^-^ | 193.05039, 178.02626, 134.03711 | *lonice raejaponicae caulis* |
| 58 | lomacarinoside B | 7.45 | 358.1257 | 417.1396 | C_16_H_22_O_9_ | -1.6 | [M+CH_3_COO]^-^ | 193.05039, 117.03448 | *lonice raejaponicae caulis* |
| 59 | Cryptochlorogenic acid | 7.51 | 354.0947 | 353.0874 | C_16_H_18_O_9_ | -1.1 | [M-H]^-^ | 307.08256, 191.05585173.04527, 135.0446893.03342 | *Forsythiae fructus* |
| 60 | UndulatosideA | 7.51 | 354.0957 | 353.0874 | C_16_H_18_O_9_ |  | [M-H]^-^ | 163.03911, 145.02853 | *Saposhnikoviae radix* |
| 61 | cis-mulberroside A | 7.68 | 568.1793 | 567.172 | C_26_H_32_O_14_ | 0.1 | [M-H]^-^ | 405.11857, 243.06595, 225.05529 | *Mori ramulus* |
| 62 | mulberroside A | 7.68 | 568.1764 | 567.172 | C_26_H_32_O_14_ | 0.1 | [M-H]^-^ | 407.13419, 245.08105, 227.06974, 135.04476 | *Mori ramulus* |
| 66 | Brevisside A | 8.15 | 478.1689 | 523.1671 | C_20_H_30_O_13_ | 0.5 | [M+HCOO]^-^ | 153.01905 | *Phellodendri chinensis cortex* |
| 67 | *β*-D-Glucopyranoside,4-(2-hydroxyethyl)phenyl6-O-D-apio-*α*-D-furanosyl | 8.15 | 432.1626 | 477.1608 | C_19_H_28_O_11_ | -1.2 | [M+HCOO]^-^ | 293.08704 | *Phellodendri chinensis cortex* |
| 69 | benzyl alcohol xylopyranosyl-(1-6)-glucopyranoside | 8.4 | 402.1521 | 447.1503 | C_18_H_26_O_10_ | -1.1 | [M+HCOO]^-^ | 207.10199 | *Forsythiae fructus* |
| 70 | phenethyl-*α*-L-rhamnopyranosyl(1→6)-*β*-D-glucopyranoside | 8.4 | 402.1521 | 447.1503 | C_18_H_26_O_10_ | -1.1 | [M+HCOO]^-^ | 207.10199, 101.02385 | *Atractylodis rhizoma* |
| 71 | phenylmethanol7-O-*β*-D-apiofuranosyl-(1→6)-*β*-D-glucopyranoside | 8.4 | 402.1521 | 447.1503 | C_18_H_26_O_10_ | -1.1 | [M+HCOO]^-^ | 207.10199, 101.02385 | *Atractylodis rhizoma* |
| 73 | (+)-lariciresinol 4,4'-O-bis-*β*-D-glucopyranoside | 9.27 | 716.2513 | 761.2501 | C_32_H_44_O_18_ | -1.1 | [M+HCOO]^-^ | 525.15995, 381.11641 | *Clematidis radix et rhizoma* |
| 74 | secoxyloganin | 9.27 | 404.1312 | 403.1239 | C_17_H_24_O_11_ | -1.6 | [M-H]^-^ | 337.09214, 191.05572, 125.02375 | *lonice raejaponicae caulis* |
| 75 | sweroside | 9.27 | 358.1257 | 403.1239 | C_16_H_22_O_9_ | -1.6 | [M+HCOO]^-^ | 125.02375 | *Cyathulae radix* |
| 76 | Butyl 5-O-caffeoylquinate | 9.4 | 410.1518 | 445.1345 | C_20_H_26_O_9_ | -1.3 | [M+Cl]^-^ | 342.1685, 179.0719 | *lonice raejaponicae caulis* |
| 78 | ailanthone | 9.83 | 376.1516 | 375.1444 | C_20_H_24_O_7_ | -1.5 | [M-H]^-^ | 360.12133, 329.10224, 283.06061, 135.04533 | *Cyathulae radix* |
| 81 | Loganin | 9.88 | 390.1524 | 435.1506 | C_17_H_26_O_10_ | -0.4 | [M+HCOO]^-^ | 341.12421, 229.10722, 197.08299, 179.0713 | *lonice raejaponicae caulis* |
| 83 | Isocorydine | 10.1 | 341.162 | 340.1547 | C_20_H_23_NO_4_ | -2.0 | [M-H]^-^ | 325.13140, 310.10746, 267.06588, 252.04205 | *Stephaniae tetrandrae radix* |
| 85 | 5-O-feruloylquinic acid | 10.68 | 368.1108 | 367.1031 | C_17_H_20_O_9_ | -1.0 | [M-H]^-^ | 177.05627, 145.02989 | *Atractylodis rhizoma, Phellodendri chinensis cortex* |
| 86 | Methyl 5-O-caffeoylquinate | 10.69 | 368.1108 | 367.1031 | C_17_H_20_O_9_ | -1.0 | [M-H]^-^ | 145.02989 | *lonice raejaponicae caulis* |
| 87 | 3-hydroxy-3-methyl-5-oxo-5-(3,4,5-trihydroxy-6-((7-hydroxy-1-(4-hydroxybenzyl)-2-methyl-1,2,3,4tetrahydroisoquinolin-8-yl)oxy)tetrahydro-2H-pyran-2-yl)methoxy)pentanoic acid | 10.82 | 591.2324 | 590.2237 | C_29_H_37_NO_12_ | -1.1 | [M-H]^-^ | 530.24373, 286.14397, 255.10167, 163.04001 | *Phellodendri chinensis cortex* |
| 88 | (7R,8S)-4,7,9,3',9'-pentahydroxy-3-methoxyl-8-4'-oxyneolignan-3'-O-*β*-D-glucopyranoside | 10.89 | 526.2046 | 571.2028 | C_25_H_34_O_12_ | -0.7 | [M+HCOO]^-^ | 313.06905, 167.07072 | *Atractylodis rhizoma* |
| 90 | Kingiside | 11.24 | 404.1316 | 403.1243 | C_17_H_24_O_11_ | -0.7 | [M-H]^-^ | 371.09848, 325.05593, 193.05054, 111.00794 | *lonice raejaponicae caulis* |
| 91 | Seguinoside B | 11.24 | 404.1316 | 403.1243 | C_17_H_24_O_11_ | -0.7 | [M-H]^-^ | 371.09848, 111.00794 | *Phellodendri chinensis cortex* |
| 92 | icariside D1[phenethyl-*β*-D-apiopyranosyl(1→6)-*β*-D-glucopyranoside] | 11.35 | 416.1677 | 461.1659 | C_19_H_28_O_10_ | -1.3 | [M+HCOO]^-^ | 329.10219, 151.03968, 125.02469 | *Atractylodis rhizoma* |
| 93 | (+)-Cycloolivil | 11.55 | 376.152 | 375.1447 | C_20_H_24_O_7_ | -0.6 | [M-H]^-^ | 329.10251, 314.07871, 283.06096, 175.03966, 161.02400, 133.02959 | *Forsythiae fructus* |
| 94 | 7-O-butylsecologanic acid | 12.13 | 430.1859 | 475.1818 | C_20_H_30_O_10_ | -0.6 | [M+HCOO]^-^ | 203.07063 | *lonice raejaponicae caulis* |
| 95 | japenol | 12.26 | 376.1518 | 375.1445 | C_20_H_24_O_7_ | -1.1 | [M-H]^-^ | 203.03433, 179.03472, 161.02445, 151.03969 | *lonice raejaponicae caulis* |
| 96 | Olivil | 12.26 | 376.1518 | 375.1445 | C_20_H_24_O_7_ | -1.1 | [M-H]^-^ | 203.03433, 179.03472, 161.02445, 151.03969 | *Forsythiae fructus* |
| 97 | 6-O-Acetyldihydroparetoside | 12.44 | 594.1947 | 639.193 | C_28_H_34_O_14_ | 0.0 | [M+HCOO]^-^ | 487.14290, 179.03472, 161.02445, 133.02930 | *Phellodendri chinensis cortex* |
| 98 | (7E)-sinapate-4-O-*β*-D-glucopyranoside | 12.53 | 386.1209 | 385.1136 | C_17_H_22_O_10_ | -1.1 | [M-H]^-^ | 203.03487, 179.03480, 161.02430, 133.02896 | *Atractylodis rhizoma* |
| 99 | coniferaldehyde glucoside | 12.53 | 340.1154 | 385.1136 | C_16_H_20_O_8_ | -1.1 | [M+HCOO]^-^ | 161.02430, 133.02896 | *Phellodendri chinensis cortex* |
| 100 | 4-Hydroxy-3,5-dimethoxycinnamic acid | 12.58 | 224.0781 | 223.0623 | C_11_H_12_O_5_ | 1.9 | [M-H]^-^ | 191.0351, 176.0101 | *Coicis semen* |
| 101 | Prim-O-glucosylcimifugin | 12.76 | 468.1627 | 513.1614 | C_22_H_28_O_11_ | 0.0 | [M+HCOO]^-^ | 359.1317, 305.1007 | *Saposhnikoviae radix* |
| 102 | Isotrilobine-N-2-oxide | 12.78 | 592.2596 | 627.229 | C_36_H_36_N_2_O_6_ | 3.6 | [M+Cl]^-^ | 359.11242 | *Stephaniae tetrandrae radix* |
| 104 | L-phenylalaninosecologanin | 13.22 | 537.2203 | 536.2136 | C_26_H_35_NO_11_ | -0.3 | [M-H]^-^ | 469.17209, 358.16045, 298.14805, 256.12954, 146.02542 | *lonice raejaponicae caulis* |
| 105 | Argentinine | 13.23 | 295.1568 | 340.155 | C_19_H_21_NO_2_ | -1.4 | [M+HCOO]^-^ | 191.05552 | *Stephaniae tetrandrae radix* |
| 106 | corypalmine | 13.23 | 341.1622 | 340.155 | C_20_H_23_NO_4_ | -1.4 | [M-H]^-^ | 312.12390, 192.10248, 177.07909, 148.07649 | *Phellodendri chinensis cortex* |
| 108 | (+)-8-hydroxyepipinores inol-4-O-*β*-D-glucopyranoside | 13.64 | 536.1893 | 535.1821 | C_26_H_32_O_12_ | -0.1 | [M-H]^-^ | 328.09381 | *Forsythiae fructus* |
| 109 | hesperidin | 13.67 | 610.1895 | 609.1825 | C_28_H_34_O_15_ | 0.0 | [M-H]^-^ | 447.15099, 179.03484, 161.02415 | *Saposhnikoviae radix, Forsythiae fructus* |
| 110 | phellavin | 13.64 | 536.1893 | 535.1821 | C_26_H_32_O_12_ | -0.1 | [M-H]^-^ | 408.14834, 269.08117, 181.05026, 166.02567 | *Phellodendri chinensis cortex* |
| 111 | glochidioboside | 13.75 | 522.2104 | 521.2026 | C_26_H_34_O_11_ | -0.4 | [M-H]^-^ | 359.14843, 344.12557, 313.10803, 241.04963, 189.05430 | *Forsythiae fructus* |
| 112 | Isoforsythiaside | 14.19 | 624.2048 | 623.1984 | C_29_H_36_O_15_ | 0.3 | [M-H]^-^ | 479.15632, 325.09195, 163.03978, 145.02974 | *Forsythiae fructus* |
| 113 | (+)-isolariciresinol-6*α*-O-*β*-D-glucopyranoside | 14.43 | 522.2099 | 567.208 | C_26_H_34_O_11_ | -0.6 | [M+HCOO]^-^ | 329.13882, 293.04469, 123.04429 | *Forsythiae fructus* |
| 114 | Liriodendrin | 14.43 | 742.2679 | 787.2661 | C_34_H_46_O_18_ | -0.7 | [M+HCOO]^-^ | 515.12022, 417.15384, 181.05031 | *lonice raejaponicae caulis* |
| 115 | calceolarioside B | 14.57 | 478.1474 | 477.1401 | C_23_H_26_O_11_ | -0.3 | [M-H]^-^ | 235.05978, 176.04686, 133.02900 | *Forsythiae fructus* |
| 118 | (+)-isolariciresinol-9-O-*β*-D-glucopyranoside | 14.73 | 522.2099 | 567.2081 | C_26_H_34_O_11_ | -0.4 | [M+HCOO]^-^ | 311.09319, 269.08143 | *Forsythiae fructus* |
| 122 | rutin | 14.8 | 610.1511 | 610.1517 | C_27_H_30_O_16_ | -3.7 | [M+e]^-^ | 301.03331 | *lonice raejaponicae caulis, Coicis semen, Forsythiae fructus* |
| 123 | (+)-lyoniresinol 9'-O-*β*-D-glucopyranoside | 15.28 | 582.2306 | 581.2233 | C_28_H_38_O_13_ | -1.1 | [M-H]^-^ | 389.15981, 359.14860, 208.07349, 165.05497 | *Clematidis radix et rhizoma* |
| 124 | phelloside | 15.43 | 696.2268 | 755.2406 | C_32_H_40_O_17_ | 0.3 | [M+CH_3_COO]^-^ | 447.09271 | *Phellodendri chinensis cortex* |
| 126 | forsythoside A | 15.59 | 624.2035 | 623.1984 | C_29_H_36_O_15_ | 0.3 | [M-H]^-^ | 609.1784, 338.0817, 311.0941 | *Forsythiae fructus* |
| 127 | forsythoside B | 15.71 | 756.2479 | 755.2407 | C_34_H_44_O_19_ | 0.3 | [M-H]^-^ | 623, 593.20855, 447.15067, 315.10812, 161 | *Forsythiae fructus* |
| 128 | forsythoside I | 15.92 | 624.2052 | 623.1987 | C_29_H_36_O_15_ | 0.8 | [M-H]^-^ | 471.14870, 325.09210, 309.09670, 163.03994, 145.02858 | *Forsythiae fructus* |
| 129 | cimifugin | 16.1 | 306.1106 | 351.1094 | C_16_H_18_O_6_ | 2.4 | [M+HCOO]^-^ | 261.0779, 243.0652 | *Saposhnikoviae radix* |
| 130 | Plantainoside A | 16.16 | 478.1471 | 477.1402 | C_23_H_26_O_11_ | 0.0 | [M-H]^-^ | 325.09210, 309.09670, 163.03994, 145.02858 | *Forsythiae fructus* |
| 133 | matairesinoside | 16.75 | 520.1953 | 519.1864 | C_26_H_32_O_11_ | -1.5 | [M-H]^-^ | 235.09880, 175.07728, 137.05984 | *Forsythiae fructus* |
| 134 | dihydrophelloside | 16.78 | 680.2304 | 519.1864 | C_26_H_32_O_11_ | -1.5 | [M-H]^-^ | 555.16327, 151.03969 | *Phellodendri chinensis cortex* |
| 135 | Isochlorogenic acid A | 16.82 | 516.1261 | 679.2231 | C_32_H_40_O_16_ | -1.8 | [M-H]^-^ | 353.08805, 191.05596, 161.02397, 135.04505 | *lonice raejaponicae caulis* |
| 136 | 3,5,6,7,8,3',4'-Heptamethoxyflavone | 16.96 | 432.1415 | 477.1397 | C_22_H_24_O_9_ | -1.1 | [M+HCOO]^-^ | 193.05093, 121.02916 | *Clematidis radix et rhizoma* |
| 137 | calceolarioside A | 16.96 | 478.147 | 477.1397 | C_23_H_26_O_11_ | -1.1 | [M-H]^-^ | 285.03855, 255.02955, 161.02409, 134.03600 | *Forsythiae fructus* |
| 138 | 3,4-O-biscaffeoylquinic acid | 17.1 | 516.1269 | 515.1197 | C_25_H_24_O_12_ | 0.3 | [M-H]^-^ | 375.06859, 353.08755, 191.05591, 173.04526 | *lonice raejaponicae caulis* |
| 139 | (+)-pinoresinol 4,4'-O-bis-*β*-D-glucopyranoside | 17.12 | 714.238 | 713.2307 | C_32_H_42_O_18_ | 1.2 | [M-H]^-^ | 515.11852, 353.08755, 337.10770 | *Clematidis radix et rhizoma* |
| 140 | Kinginoside | 17.22 | 680.2318 | 679.2231 | C_32_H_40_O_16_ | -1.8 | [M-H]^-^ | 555.16327, 151.03969 | *lonice raejaponicae caulis* |
| 142 | 5-O-Methylvisammioside | 17.84 | 452.1679 | 497.1661 | C_22_H_28_O_10_ | -0.7 | [M+HCOO]^-^ | 431.1902, 349.1849 | *Saposhnikoviae radix* |
| 145 | 3,4-dihydroxy-allylbenzene | 18.35 | 150.06808 | 149.0606 | C_9_H_10_O_2_ | -0.6 | [M-H]^-^ | 123.0423, 116.9257 | *Forsythiae fructus* |
| 146 | decursin | 18.35 | 328.1305 | 373.1287 | C_19_H_20_O_5_ | -1.5 | [M+HCOO]^-^ | 267.06554, 151.04004, 147.04495, 129.03229 | *Saposhnikoviae radix* |
| 147 | forsythialan A | 18.35 | 374.136 | 373.1287 | C_20_H_22_O_7_ | -1.5 | [M-H]^-^ | 343.11373, 151.04004 | *Forsythiae fructus* |
| 148 | Clemomanshurinane B | 18.77 | 474.1898 | 519.188 | C_25_H_30_O_9_ | 1.6 | [M+HCOO]^-^ | 357.13427, 342.11178, 235.06079, 122.03856 | *Clematidis radix et rhizoma* |
| 149 | 1,3-di-O-caffeoylquinic acid | 18.82 | 516.1272 | 515.1199 | C_25_H_24_O_12_ | 0.8 | [M-H]^-^ | 353.08805, 191.05596, 173.04582, 135.04505, 93.03385 | *Atractylodis rhizoma* |
| 153 | 7'-epi-8-hydroxypinoresinol | 19.15 | 374.1367 | 373.129 | C_20_H_22_O_7_ | -0.6 | [M-H]^-^ | 137.06018, 122.03944 | *Forsythiae fructus* |
| 154 | Resveratrol | 19.15 | 228.0794 | 227.0721 | C_14_H_12_O_3_ | 3.4 | [M-H]^-^ | 199.0973, 179.0349, 155.1091 | *Mori ramulus* |
| 155 | Ledebouriellol | 19.15 | 374.1363 | 373.129 | C_20_H_22_O_7_ | -0.6 | [M-H]^-^ | 207.06625, 193.05051, 161.02404, 149.06063 | *Saposhnikoviae radix* |
| 157 | (+)-epipinoresinol-4'-O-*β*-D-glucopyranoside | 19.28 | 520.1953 | 519.1864 | C_26_H_32_O_11_ | -1.5 | [M-H]^-^ | 235.09880, 175.07728 | *Forsythiae fructus* |
| 158 | clemomanshurinane A | 19.28 | 474.1894 | 519.1876 | C_25_H_30_O_9_ | 0.7 | [M+HCOO]^-^ | 357.13386, 342.10994, 337.10766, 221.08147, 122.03728 | *Clematidis radix et rhizoma* |
| 161 | grandifloroside | 19.69 | 538.1687 | 537.1614 | C_25_H_30_O_13_ | 0.1 | [M-H]^-^ | 479.15907, 375.12908, 201.01647, 161.02462, 135.04512, 121.02882 | *lonice raejaponicae caulis* |
| 162 | 7S,8R-4,7,9,9'-tetxohydroxy-3,3'-dimethoxy-8-O-4-nelligan | 19.95 | 378.1678 | 423.166 | C_20_H_26_O_7_ | -0.1 | [M+HCOO]^-^ | 343.11754, 179.03485, 145.02935, 135.04453, 117.03400 | *Phellodendri chinensis cortex* |
| 164 | sec-o-glucosylhamaudol | 21.08 | 438.152 | 483.1502 | C_21_H_26_O_10_ | -1.3 | [M+HCOO]^-^ | 437.1448, 312.1240, 275.0939 | *Saposhnikoviae radix* |
| 165 | forsythialanside E | 21.81 | 522.1737 | 521.1665 | C_25_H_30_O_12_ | 0.0 | [M-H]^-^ | 359.11273, 221.08399, 163.03985, 145.02951 | *Forsythiae fructus* |
| 166 | Ethyl caffeate | 21.83 | 208.0742 | 207.0669 | C_11_H_12_O_4_ | -0.4 | [M-H]^-^ | 116.9279 | *Phellodendri chinensis cortex\|\|lonice raejaponicae caulis* |
| 167 | (+)-1-hydroxypinoresinol-4''-O-*β*-D-glucopyranosi-de | 22.03 | 534.2103 | 579.2083 | C_27_H_34_O_11_ | 0.0 | [M+HCOO]^-^ | 356.12590, 121.02880 | *Forsythiae fructus* |
| 168 | syringaresinol-4'-O-*β*-D-glucopyranoside | 22.03 | 580.2157 | 579.2085 | C_28_H_36_O_13_ | 0.3 | [M-H]^-^ | 371.14929, 356.12590, 121.02880 | *Atractylodis rhizoma* |
| 171 | (+)-pinoresinol monomethyl ether-4-O-*β*-D-glucoside | 22.19 | 534.2096 | 579.2083 | C_27_H_34_O_11_ | 0.0 | [M+HCOO]^-^ | 355.15246, 189.09117 | *Forsythiae fructus* |
| 172 | 6'-O-p-apiduranosylsweroside | 22.39 | 504.1867 | 504.1872 | C_22_H_32_O_13_ | 4.7 | [M+e]^-^ | 461.16324, 275.09540 | *lonice raejaponicae caulis* |
| 173 | cnidimoide A | 22.65 | 438.1524 | 483.1504 | C_21_H_26_O_10_ | -0.7 | [M+HCOO]^-^ | 315.1584, 275.0939 | *Saposhnikoviae radix* |
| 174 | 4'-O-*β*-D-glucopyranosylvisamminol | 22.66 | 438.1524 | 483.1504 | C_21_H_26_O_10_ | -0.7 | [M+HCOO]^-^ | 275.0939, 257.0811 | *Saposhnikoviae radix* |
| 176 | Ethyl 4-hydroxyphenylacetate | 22.9 | 180.0793 | 179.0721 | C_10_H_12_O_3_ | -1.2 | [M-H]^-^ | 116.9279 | *Forsythiae fructus* |
| 178 | oleanolic acid 3-O-*β*-D-glucopyranosyl-(1,4)-*β*-D-glucopyranosyl-(1,4)-*β*-D-ribopyranosyl-(1,3)-ɑ-L-rhamnopyranosyl-(1,2)-ɑ-L-arabinopyranoside | 23.3 | 1220.6251 | 1255.5945 | C_59_H_96_O_26_ | 4.9 | [M+Cl]^-^ | 899.46321, 881.48808, 659.33021 | *Clematidis radix et rhizoma* |
| 179 | sarsasaponin | 23.32 | 1048.5444 | 1093.5426 | C_51_H_84_O_22_ | -0.9 | [M+HCOO]^-^ | 901.47948, 883.46902, 755.42155, 593.36864, | *Cyathulae radix* |
| 181 | 6'-O-*β*-D-glucosylgentiopicroside | 23.51 | 432.1057 | 491.1196 | C_21_H_20_O_10_ | 0.2 | [M+CH3COO]^-^ | 367.1200 | *Cyathulae radix* |
| 182 | Isorhamnetin-3-O-*β*-D-Glucoside | 23.51 | 478.1109 | 477.1039 | C_22_H_22_O_12_ | 0.1 | [M-H]^-^ | 317.06579 | *lonice raejaponicae caulis* |
| 183 | naringenin | 23.51 | 272.0688 | 271.0615 | C_15_H_12_O_5_ | 0.1 | [M-H]^-^ | 227.1287, 161.0233 | *Clematidis radix et rhizoma* |
| 184 | (+)-Pinoresinol | 23.86 | 358.141 | 357.1337 | C_20_H_22_O_6_ | -2.0 | [M-H]^-^ | 137.06018, 122.03944 | *lonice raejaponicae caulis* |
| 185 | 4-ketopinol ester | 23.86 | 358.1409 | 357.1337 | C_20_H_22_O_6_ | -2.0 | [M-H]^-^ | 121.02917 | *Coicis semen* |
| 186 | 4'-preneyl oxyresveratrol | 23.86 | 312.1355 | 357.1337 | C_19_H_20_O_4_ | -2.0 | [M+HCOO]^-^ | 121.02917 | *Mori ramulus* |
| 187 | rengyoside A | 23.86 | 322.1643 | 357.1337 | C_14_H_26_O_8_ | 4.2 | [M+Cl]^-^ | 205.07243, 163.06130 | *Forsythiae fructus* |
| 188 | huzhangoside B | 24.24 | 1336.6617 | 1335.6586 | C_64_H_104_O_29_ | -0.3 | [M-H]^-^ | 925.44407, 353.22921 | *Clematidis radix et rhizoma* |
| 189 | clematichinenosideC | 24.6 | 1498.7177 | 1497.7105 | C_70_H_114_O_34_ | -1.0 | [M-H]^-^ | 1335.65847, 1027.54846, 455.35224, 289.09233 | *Clematidis radix et rhizoma* |
| 190 | 11,12,15-Trihydroxy-13-en-oc-tadecenoic acid | 24.92 | 330.241 | 329.2335 | C_18_H_34_O_5_ | 0.5 | [M-H]^-^ | 213.15043 | *lonice raejaponicae caulis* |
| 191 | saponin CP8 | 24.94 | 1016.5208 | 1075.5347 | C_50_H_80_O_21_ | 1.5 | [M+CH_3_COO]^-^ | 737.41294 | *Clematidis radix et rhizoma* |
| 192 | isopimpinellin | 25.03 | 246.054 | 245.0427 | C_13_H_10_O_5_ | -2.9 | [M-H]^-^ | 164.1084 | *Saposhnikoviae radix* |
| 193 | pinelliae palmatum L | 25.04 | 330.241 | 329.2335 | C_18_H_34_O_5_ | 0.5 | [M-H]^-^ | 213.15043, 195.13803 | *Cyathulae radix* |
| 194 | saponins CP7 | 25.1 | 1000.5248 | 1045.523 | C_50_H_80_O_20_ | 0.5 | [M+HCOO]^-^ | 899.46467, 883.47055, 737.41215, 575.35915, 179.05622 | *Clematidis radix et rhizoma* |
| 195 | Loniceroside B | 25.21 | 1190.6089 | 1249.6228 | C_58_H_94_O_25_ | 0.4 | [M+CH_3_COO]^-^ | 1043.54453, 833.47010, 737.41162, 469.15597 | *lonice raejaponicae caulis* |
| 196 | saponins CP4 | 25.24 | 838.4717 | 883.4699 | C_44_H_70_O_15_ | 0.2 | [M+HCOO]^-^ | 737.41162, 575.3591 | *Clematidis radix et rhizoma* |
| 197 | rhamnetin | 25.38 | 316.058 | 315.0507 | C_16_H_12_O_7_ | -1.1 | [M-H]^-^ | 299.01897, 165.01880 | *Forsythiae fructus* |
| 198 | hederagenin 3-O-*β*-D-glucopyranosyl-(1,4)-*β*-D-xylopyranosyl-(1,3)-ɑ-L-rhamnopyranosyl-(1,2)-ɑ-L-arabinopyranoside | 25.5 | 1044.5506 | 1089.5488 | C_52_H_84_O_21_ | 0.1 | [M+HCOO]^-^ | 1029.52814, 865.46264, 733.45295 | *Clematidis radix et rhizoma* |
| 199 | Loniceracetalide A | 25.61 | 460.1951 | 519.209 | C_21_H_32_O_11_ | 1.2 | [M+CH3COO]^-^ | 109.029 | *lonice raejaponicae caulis* |
| 200 | Loniceroside A | 25.64 | 1044.5506 | 1089.5488 | C_52_H_84_O_21_ | 0.1 | [M+HCOO]^-^ | 1029.52814, 897.48557, 883.47076, 865.46264, 733.45295 | *lonice raejaponicae caulis* |
| 201 | Formononetin | 25.65 | 268.0739 | 327.0877 | C_16_H_12_O_4_ | 1.0 | [M+CH_3_COO]^-^ | 109.029 | *Clematidis radix et rhizoma* |
| 202 | morusignin A | 25.65 | 328.095 | 327.0877 | C_18_H_16_O_6_ | 1.0 | [M-H]^-^ | 109.029 | *Mori ramulus* |
| 203 | Phellodendron acid | 25.87 | 470.1949 | 515.1923 | C_26_H_30_O_8_ | 0.0 | [M+HCOO]^-^ | 161.06102 | *Phellodendri chinensis cortex* |
| 204 | 7*α*,10*α*-epoxyguaiane-4*α*,11-diol | 25.98 | 270.2189 | 329.2335 | C_16_H_30_O_3_ | 0.5 | [M+CH_3_COO]^-^ | 119.04891 | *Atractylodis rhizoma* |
| 205 | Isobutylparaben | 26.28 | 194.0948 | 193.0876 | C_11_H_14_O_3_ | -0.9 | [M-H]^-^ | 164.9986 | *Forsythiae fructus* |
| 206 | panax notoginsenoside IV A methyl ester | 26.3 | 794.4444 | 793.4371 | C_42_H_66_O_14_ | -1.1 | [M-H]^-^ | 567.36868, 175.02424 | *Cyathulae radix* |
| 207 | 11(Z)-hexadecenoic Acid | 26.99 | 254.2242 | 313.238 | C_16_H_30_O_2_ | -1.2 | [M+CH_3_COO]^-^ | 125.09789 | *Clematidis radix et rhizoma* |
| 208 | tormentic acid | 27.02 | 488.3497 | 487.3424 | C_30_H_48_O_5_ | -1.0 | [M-H]^-^ | 333.2062 | *Clematidis radix et rhizoma* |
| 209 | mulberrin | 27.08 | 422.172 | 421.1652 | C_25_H_26_O_6_ | -1.0 | [M-H]^-^ | 311.05468, 241.04931 | *Mori ramulus* |
| 210 | 25R-inokosterone | 27.56 | 480.3086 | 480.3092 | C_27_H_44_O_7_ | -0.2 | [M+e]^-^ | 255.23261 | *Cyathulae radix* |
| 211 | ecdysterone | 27.56 | 480.3086 | 480.3092 | C_27_H_44_O_7_ | -0.2 | [M+e]^-^ | 255.23261 | *Cyathulae radix* |
| 213 | hamaudol | 27.9 | 276.1004 | 275.096 | C_15_H_16_O_5_ | 2.6 | [M-H]^-^ | 259.1003 | *Stephaniae tetrandrae radix* |
